# Characterization of CD90/Thy-1 as a crucial molecular signature for myogenic differentiation in human urine-derived cells through single-cell RNA sequencing

**DOI:** 10.1038/s41598-024-52530-5

**Published:** 2024-01-28

**Authors:** Katsuhiko Kunitake, Norio Motohashi, Takafumi Inoue, Yutaka Suzuki, Yoshitsugu Aoki

**Affiliations:** 1 Department of Molecular Therapy, National Institute of Neuroscience, National Center of Neurology and Psychiatry (NCNP), 4-1-1 Ogawa-Higashi, Kodaira, Tokyo, 187-8502 Japan; 2 Department of Life Science and Medical Bioscience, Waseda University, Tokyo, Japan; 3 Laboratory of Systems Genomics, Department of Computational Biology and Medical Sciences, Graduate School of Frontier Sciences, The University of Tokyo, Tokyo, Japan; 4 Department of NCNP Brain Physiology and Pathology, Graduate School of Medical and Dental Sciences, Tokyo Medical and Dental University, Tokyo, Japan

**Keywords:** Cell biology, Drug discovery, Molecular biology, Stem cells

## Abstract

Human urine-derived cells (UDCs) are primary cultured cells originating from the upper urinary tract and are known to be multipotent. We previously developed *MYOD1*-transduced UDCs (MYOD1-UDCs) as a model recapitulating the pathogenesis of Duchenne muscular dystrophy (DMD) caused by a lack of dystrophin. MYOD1-UDCs also allow evaluation of the efficacy of exon skipping with antisense oligonucleotides. However, despite the introduction of *MYOD1*, some MYOD1-UDCs failed to form myotubes, possibly because of heterogeneity among UDCs. Here, we carried out single-cell RNA-sequencing analyses and revealed that CD90/Thy-1 was highly expressed in a limited subpopulation of UDCs with high myogenic potency. Furthermore, CD90-positive MYOD1-UDCs, but not CD90-negative cells, could form myotubes expressing high levels of myosin heavy chain and dystrophin. Notably, overexpression of CD90 in CD90-negative MYOD1-UDCs did not enhance myogenic differentiation, whereas CD90 suppression in CD90-positive UDCs led to decreased myotube formation and decreased myosin heavy chain expression. CD90 may thus contribute to the fusion of single-nucleated MYOD1-UDCs into myotubes but is not crucial for promoting the expression of late muscle regulatory factors. Finally, we confirmed that CD90-positive MYOD1-UDCs derived from patients with DMD were a valuable tool for obtaining a highly reproducible and stable evaluation of exon skipping using antisense oligonucleotide.

## Introduction

Understanding the pathophysiology of human neuromuscular diseases is crucial for drug development. Although relevant studies are commonly conducted in animal models, including mice, it is necessary to consider the genetic and physiological differences between humans and animals when studying the pathologic mechanisms and therapeutic effects^1^. In this context, cellular models of human neuromuscular diseases may bring us closer to achieving personalized therapy for individual patients.

The appropriate cells for studies examining the pathogenesis of neuromuscular diseases or drug development remain controversial. Urine-derived cells (UDCs) are human primary cultured cells that are thought to originate from the kidney and/or the upper urinary tract^2^. In 1972, Sutherland and Bain first reported a method for isolating UDCs from the urine of newborn children^3^. UDCs have since been reported to be able to self-renew and differentiate into several cell types, including endothelial cells, uroepithelial cells, smooth muscle cells, neural stem cells, and skeletal muscle cells^4^. Moreover, UDCs show high proliferative ability and telomerase activity compared with other widely used stem cells such as bone marrow stem cells, blood progenitor cells, keratinocyte progenitor cells, umbilical cord stem cells, and adipose-derived stem cells^2,5–7^. Furthermore, UDCs express high levels of mesenchymal stem cell (MSC) markers, including CD44, CD73, CD29, CD105, CD166, CD90, and CD13^8^, and pluripotent stem cell markers, including POU5F1 or Oct 3/4, c-Myc, SSEA-1/4, and Klf-4^9^. The use of UDCs has advantages, including relatively straightforward, non-invasive, and repeatable methods of isolation^10^.

Several studies on skeletal muscle diseases indicated that UDCs could be induced to differentiate into myogenic-lineage cells by direct reprogramming via MyoD1 as a muscle master regulatory factor (MRF). Falzarano et al. demonstrated that UDCs derived from patients with Duchenne muscular dystrophy (DMD) retained patient-specific DMD mutations and that *MYOD1*-transduced UDCs (MYOD1-UDCs) showed no dystrophin expression^11^. They additionally confirmed that truncated dystrophin was restored by in-framing with antisense oligonucleotides (ASOs) against exon 44 of DMD^11^. We previously reported a novel technology to apply patient-derived MYOD1-UDCs as a primary myoblast model of DMD^12^. In that study, 3-deazaneplanocin A hydrochloride (DZNep), which inhibits the histone methylating enzyme EZH2, promoted the differentiation of MYOD1-UDCs into myotubes. Moreover, an in vitro assay using these DMD patient-derived myotubes showed that ASO drugs increased expression levels of the truncated dystrophin protein in a dose-dependent manner. Direct reprogramming of UDCs could thus potentially be used to study the pathophysiology of muscular diseases, diagnose, and develop novel therapies.

However, our previous study also showed that although some UDCs that overexpressed MyoD1 could differentiate into muscle cells, others did not, despite treatment with DZnep^12^. Specifically, some MYOD1-UDCs differentiated into myogenin- and myosin heavy chain (MyHC)-positive multinucleated myotubes, while others remained myogenin- and MyHC-negative single nucleated cells. We hypothesized that this reflected the existence of specific subpopulations of cells with high myogenic potency among heterogeneous UDCs.

Here, we aimed to clarify a crucial molecular signature for myogenic differentiation in UDCs through single-cell RNA sequencing (RNA-seq).

Combined with fluorescence-activated cell sorting (FACS), we successfully established a highly efficient protocol for differentiating MYOD1-UDCs into mature myotubes, which is expected to have applications for evaluating therapeutics for various muscular diseases.

## Results

### UDCs comprised heterogeneous cell subpopulations based on RNA-seq profiling

**Figure 1:**
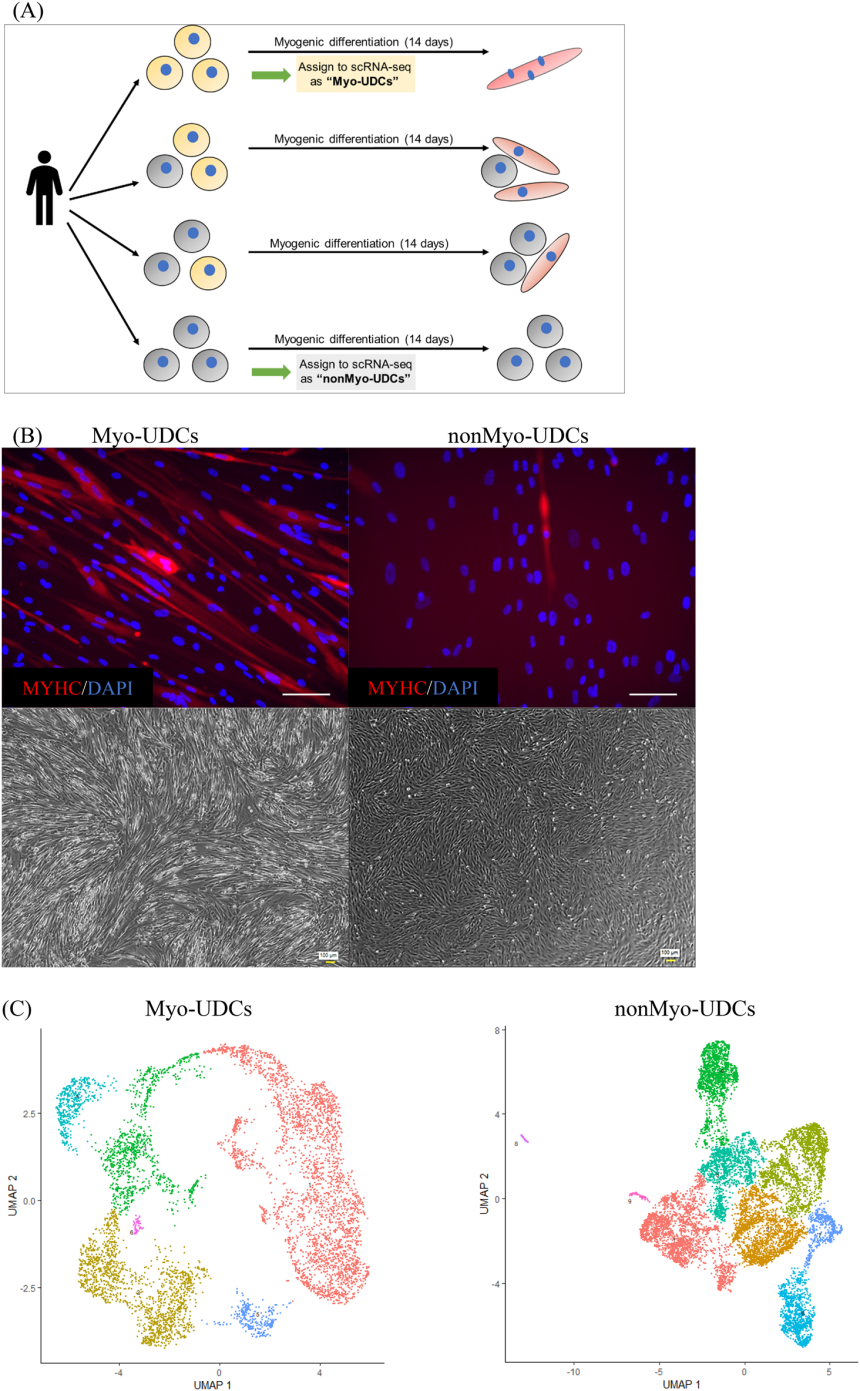

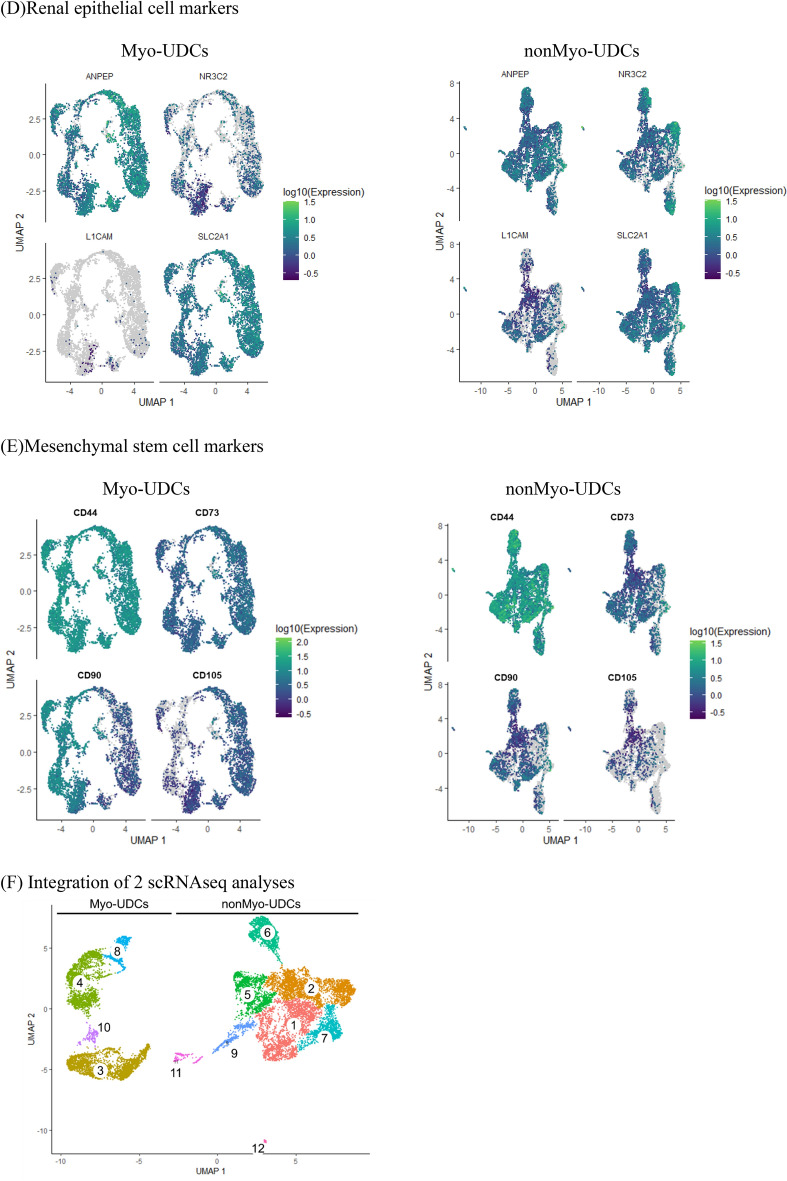

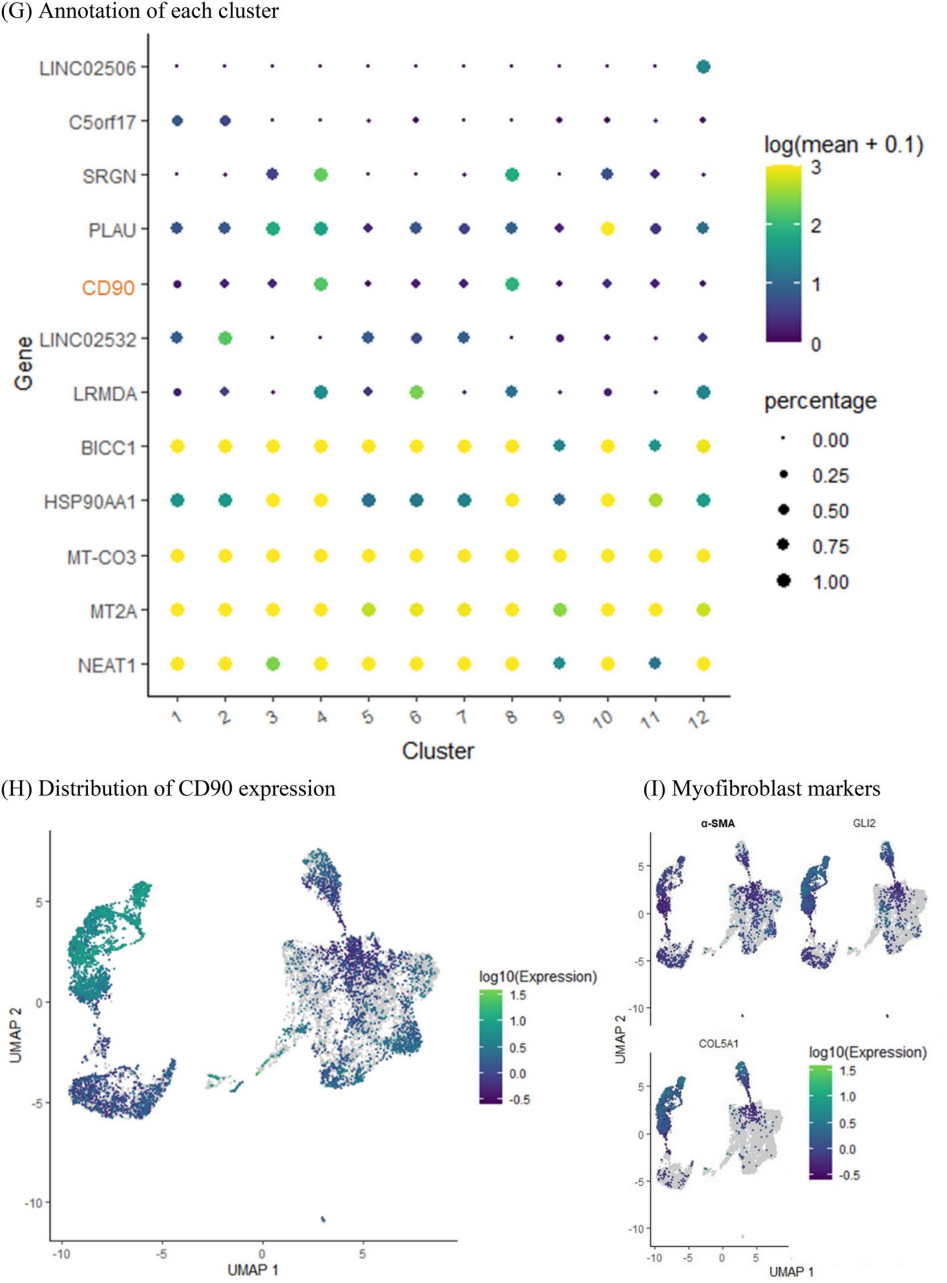
ScRNA-seq analyses revealed cellular heterogeneity of human UDCs and identified CD90 as a candidate marker for myogenic differentiation. (**A**) Selection of UDC samples for scRNA-seq. (**B**) Representative images of Myo-UDCs and nonMyo-UDCs. Phase-contrast and immunocytochemistry for MyHC (red) at 14th day after differentiation. Blue, DAPI staining. Scale bar denotes 100 µm. (**C**) Transcriptomic atlas of all cells dissociated from Myo-UDCs and nonMyo-UDCs. Single-cell expression of (**D**) kidney and (**E**) MSC markers. (**F**) Integrated transcriptomic atlas generated from Myo-UDCs and nonMyo-UDCs. (**G**) List of marker genes expressed by each cluster in integrated scRNA-seq. (**H**) Distribution of CD90 expression in integrated scRNA-seq.

We initially repeatedly isolated UDCs from the same healthy donor and transduced the established UDCs with *MYOD1* using a retroviral vector before inducing myogenic differentiation (Fig. 1A). We evaluated the efficiency of myogenic differentiation of MYOD1-UDCs by staining for MyHC at 14 days post-induction. We identified two types of UDCs, "Myo-UDCs" and "nonMyo-UDCs", with high and low myogenic potencies, respectively (Fig. 1B). To distinguish between these, we performed time-lapse imaging analysis using cultured UDCs (Movie 1), based on a previous study, which indicated that differences in the abilities of UDCs could be distinguished by their shape^13^. We accordingly observed, "rice-shaped" (RS-UDCs) and "spindle-shaped" UDCs (SS-UDCs) within the same plates; however, continuous observation revealed that the differently shaped UDCs moved around the plates and changed their morphology from rice to spindle or from spindle to rice (Movie 1). These results suggested that cell morphology alone cannot be used to distinguish between UDCs with high or low myogenic potential, and there is, thus, a need to identify potential markers to purify and isolate Myo-UDCs.

We further investigated the heterogeneity of UDCs by single-cell RNA-seq (scRNA-seq) of Myo-UDCs and nonMyo-UDCs (Fig. 1C). Both cell populations comprised multiple clusters. Kidney cell and MSC marker expression levels varied among the clusters (Fig. 1D,E). Notably, L1CAM expression levels were lower in Myo-UDCs than in nonMyo-UDCs (Fig. 1D). In contrast, both Myo-UDCs and nonMyo-UDCs expressed CD44 and CD73 homogeneously in every cluster (Fig. 1E). To find a difference between the two samples, we integrated two scRNA-seq analyses (Fig. 1F). We showed that Myo-UDCs included specific clusters expressing high levels of CD90 (Fig. 1G,H). Moreover, several myofibroblast markers including a-SMA were expressed mainly on the CD90-positive UDCs in the map (Fig. 1I).

### CD90-positive UDCs had high potency to differentiate into myotubes by inducing MYOD1

**Figure 2:**
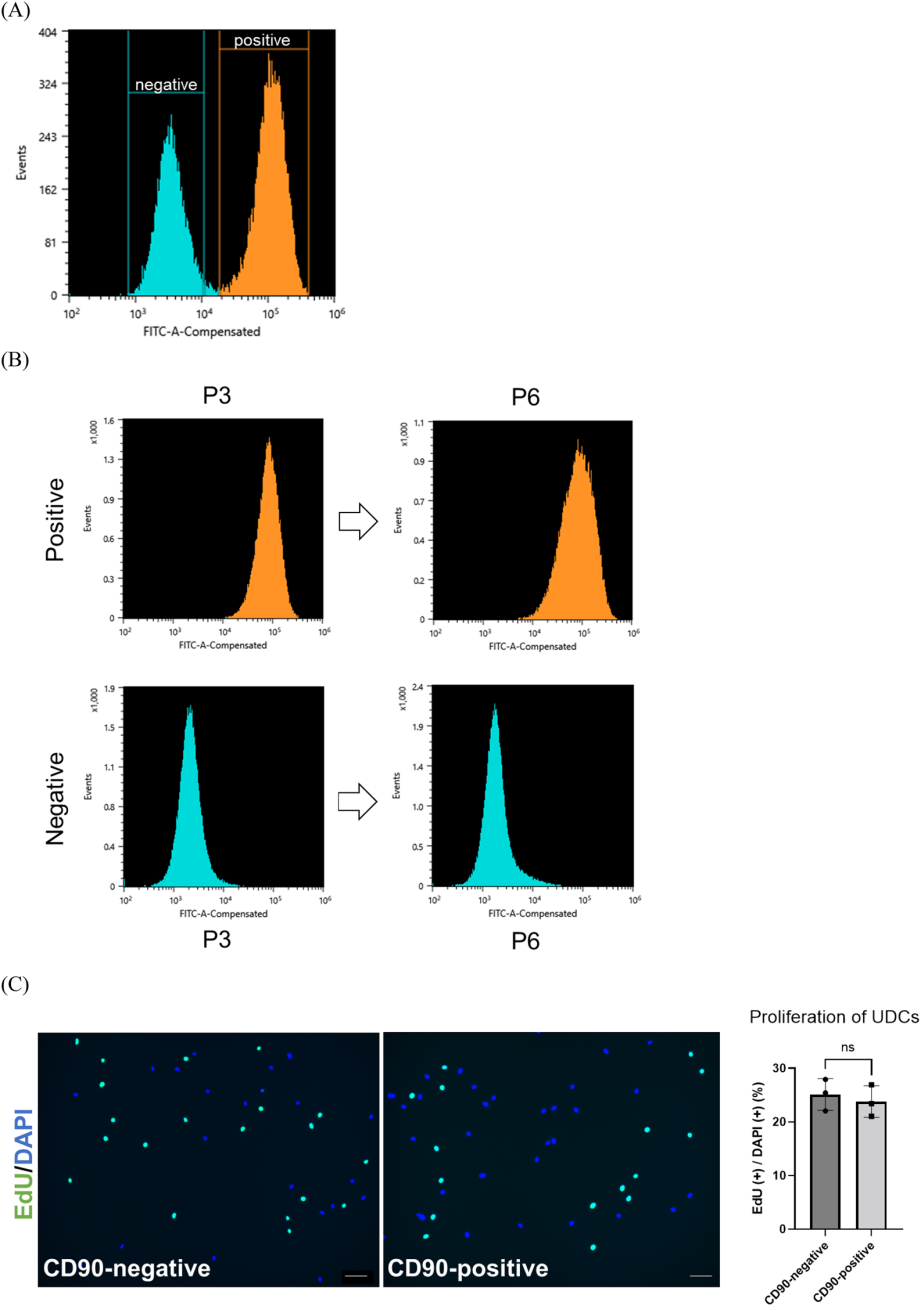

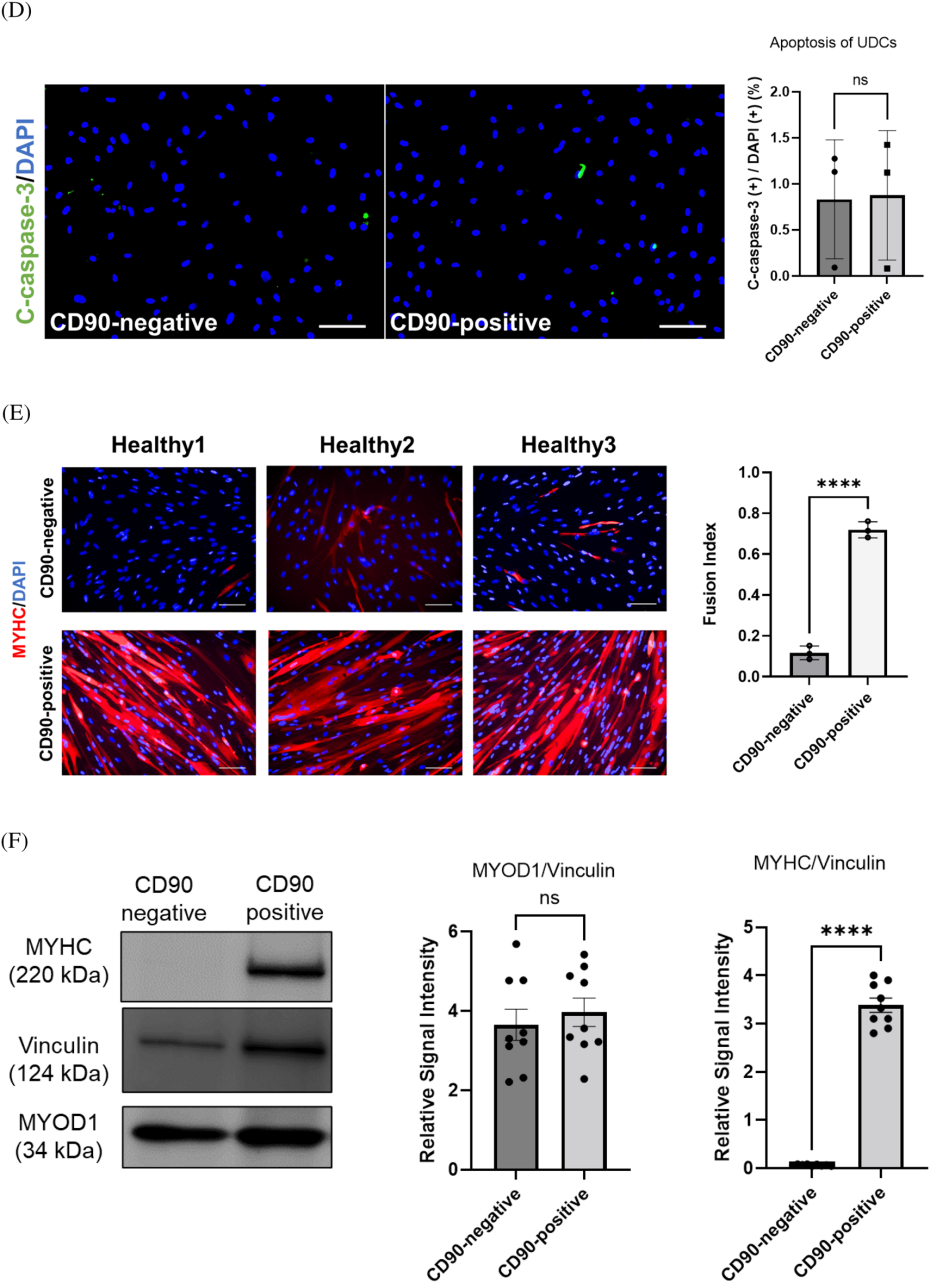

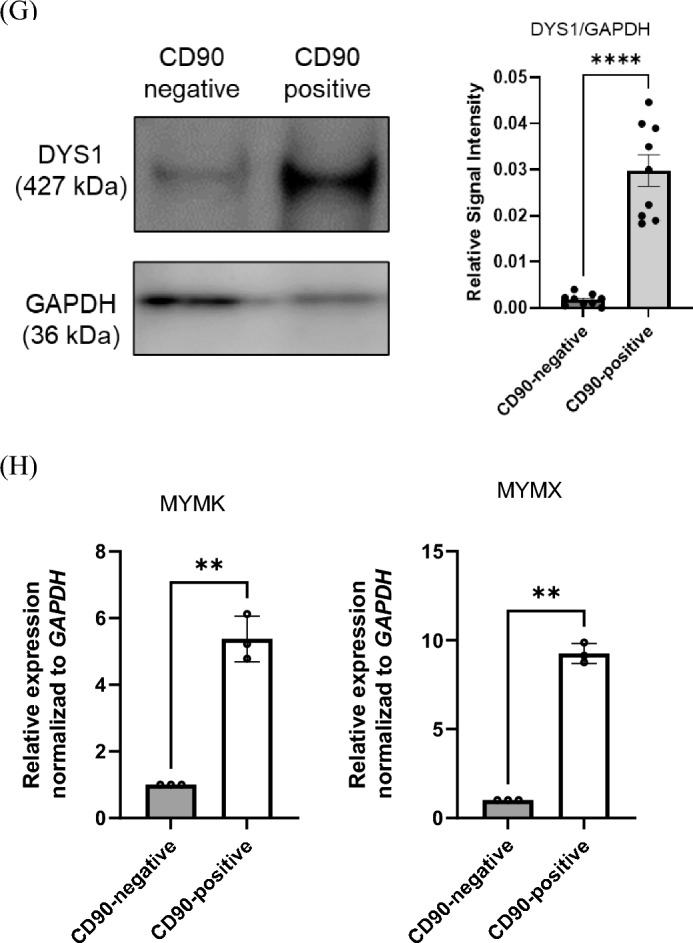
CD90-positive UDCs showed high potency for myotube differentiation after *MYOD1* gene induction. (**A**) Representative flow cytometric analysis of CD90 in UDCs at 3rd passage. (**B**) Representative image of follow-up flow cytometric analysis in CD90-positive and CD90-negative UDCs. (**C**) EdU assay to evaluate proliferative ability of UDCs. Green, EdU; blue, DAPI staining. Scale bar denotes 100 µm; n = 3 per group. Two-tailed t-test was used for comparison. Data expressed as mean ± standard error (SEM). (**D**) Immunocytochemical detection of c-casp3 (green) in UDCs demonstrating apoptotic ability. Blue, DAPI staining. Scale bar denotes 100 µm; n = 3 per group. Two-tailed t-test was used for comparison. Data expressed as mean ± SEM. (**E**) Immunocytochemical detection of MyHC (red) in UDCs. Blue, DAPI staining. Scale bar denotes 100 µm. Fusion index was calculated as percentage of nuclei within MyHC-positive myotubes in nine randomly selected images from three healthy individuals. Two-tailed t-test was used for comparisons. Data expressed as mean ± SEM; ****P < 0.0001. (**F**, **G**) Immunoblotting of MyHC, MyoD1, and dystrophin (DYS1) in MYOD1-UDCs on 14th day after differentiation. Anti-Vinculin and GAPDH antibodies were used as a loading control. Original blots are presented in Supplementary Figures S5A and B. Two-tailed t-test was used for comparisons. Data expressed as mean ± SEM; *P < 0.05, ****P < 0.0001. (**H**) Quantitative reverse transcription (qRT)-PCR analysis of *MYMK* and *MYMX* expression on 3rd day after differentiation; n = 3 each. Two-tailed t-test was used for statistical analysis. Data expressed as mean ± SEM; **P < 0.01.

Based on the scRNA-seq data, we considered CD90/Thy-1 a candidate marker of a UDC subpopulation with high potency for myogenic differentiation. To confirm this result, cultured UDCs were stained with CD90-antibody and analyzed by FACS. As expected, UDCs consisted of CD90-positive and CD90-negative cells (Fig. 2A). When we performed the vitality test for our UDCs, most cells were 7AAD negative regardless of their CD90 expression (Fig. S1). We traced the temporal profile of the CD90-positive/negative ratio of UDCs by examining both CD90-positive and CD90-negative UDC samples by FACS at different passages, including third and sixth passage cells (Fig. 2B), and found that CD90-positive and CD90-negative UDCs maintained their CD90 expression characteristics consistently during passage in vitro.

We evaluated the abilities of UDCs by performing cell proliferation assays using EdU (Fig. 2C) and manual calculation at several time points (Fig. S2). As a result, we found that the numbers of proliferating cells were comparable between CD90-positive and CD90-negative UDCs. CD90-positive and -negative MYOD1-UDCs were also cultured under starved conditions to assess apoptosis resistance by staining with an anti-cleaved-caspase 3 (c-casp3) antibody. UDCs cultured under PBS showed positive staining for c-casp3 (Figure S3A). In contrast, the number of c-casp3-positive cells was significantly limited after serum starvation, with no significant differences among them (Fig. 2D). However, the fusion index reflecting the number of MyHC-stained multinucleated myofibers was significantly higher in CD90-positive MYOD1-UDCs compared with CD90-negative cells at 14 days after the induction of myogenic differentiation (Fig. 2E). We confirmed this result by immunoblotting of MyoD1 and MyHC proteins in differentiated MYOD1-UDCs at Day 14. The MyoD1 expression levels were similar in CD90-positive and CD90-negative MYOD1-UDCs, but MyHC expression levels were higher in CD90-positive than CD90-negative cells (Fig. 2F and S4A). Consistent with the expression levels of MyHC, CD90-positive MYOD1-UDC myotubes also showed relatively high dystrophin expression levels compared with CD90-negative cells (Fig. 2G and S4B). The immunoblotting analysis regarding dystrophin was confirmed by NCL-DYS1 antibody. Just in case, we checked the transduction efficiency of the *MYOD1*-retroviral vector into both CD90-positive and negative cells (Fig. S5). As a result, MyoD was transduced into approximately 70% of the UDCs regardless of their CD90 expression. Moreover, as a reference, we performed quantitative polymerase chain reaction (qPCR) experiments of fusion-related genes, including Myomaker (*MYMK*) and Myomixer (*MYMX*), in CD90-positive and negative MYOD1-UDCs (Fig. 2H). CD90-positive cells showed higher expression of *MYMK* and *MYMX* compared with CD90-negative cells. We speculated that CD90-positive UDCs possessed high potency for myogenic differentiation after inducing *MYOD1* gene among heterogeneous UDCs. Intriguingly, repeated qPCR showed that *CD90* expression decreased during the differentiation of CD90-positive MYOD1-UDCs to myotubes (Fig. S6).

### CD90-overexpression did not promote myotube formation in CD90-negative MYOD1-UDCs

**Figure 3:**
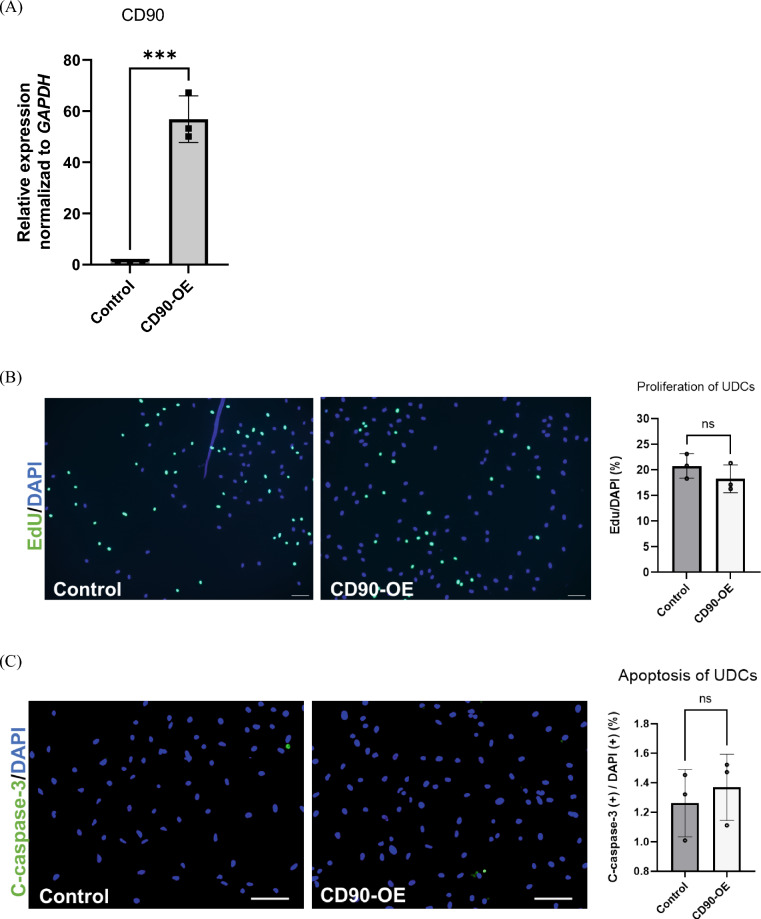

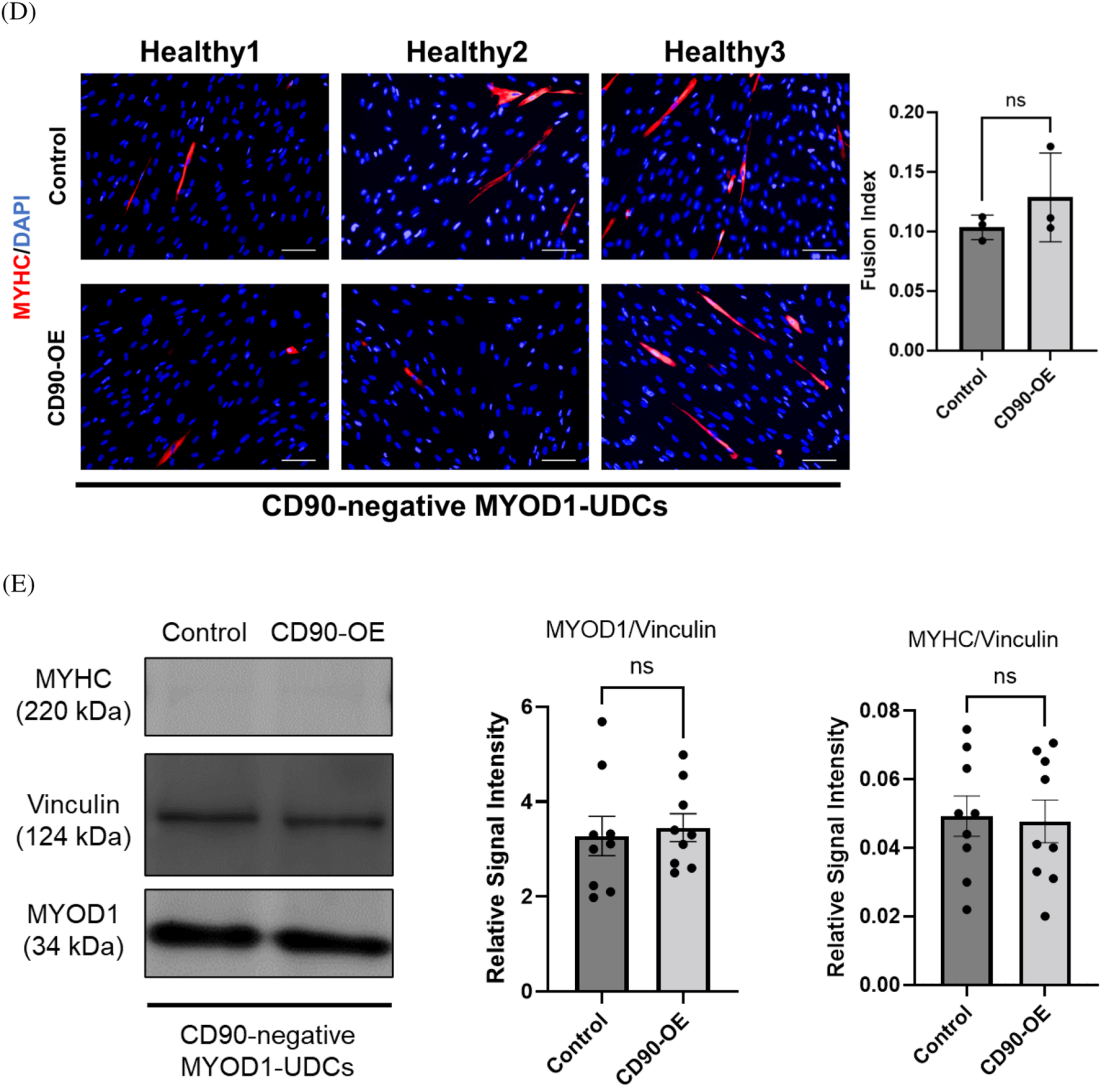
*CD90*-overexpression failed to promote myotube formation in CD90-negative MYOD1-UDCs. (**A**) Expression of CD90 in CD90-negative MYOD1-UDCs determined by qRT-PCR; n = 3 each. Two-tailed t-test was used for statistical analysis. Data expressed as mean ± SEM; ***P < 0.001. (**B**) EdU assay to evaluate proliferative ability of CD90-negative MYOD1-UDCs. Green, EdU; blue, DAPI staining. Scale bar denotes 100 µm; n = 3 per group. Two-tailed t-test was used for statistical analysis. Data expressed as mean ± SEM. (**C**) Immunocytochemical detection of c-casp3 (green) in CD90-negative MYOD1-UDCs to evaluate apoptotic ability. Blue, DAPI staining. Scale bar denotes 100 µm; n = 3 per group. Two-tailed t-test was used for statistical analysis. Data expressed as mean ± SEM. (**D**) Immunocytochemical detection of MyHC (red) in CD90-negative MYOD1-UDCs. Blue, DAPI staining. Scale bar denotes 100 µm. Fusion index was calculated as percentage of nuclei within MyHC-positive myotubes in nine randomly selected images from three healthy individuals. Two-tailed t-test was used for comparison. Data expressed as mean ± SEM. (**E**) Immunoblotting of MyHC and MyoD1 in CD90-negative MYOD1-UDCs on 14th day after differentiation. Anti-Vinculin antibody was used as a loading control. Original blots are presented in Supplementary Figure S5A. Two-tailed t-test was used for comparisons. Data expressed as mean ± SEM.

To determine if CD90 expression functionally affected myogenic differentiation in MYOD1-UDCs, we used a lentiviral vector to overexpress CD90 in CD90-negative MYOD1-UDCs as a control. We confirmed that *CD90* expression was effectively increased in the cells (Fig. 3A). Cell proliferation assays showed no difference in the number of EdU-stained proliferating between the control and CD90-overexpressing (OE) MYOD1-UDCs (Fig. 3B). Moreover, CD90-OE MYOD1-UDCs were cultured under starved conditions and stained with a c-casp3 antibody. The number of c-casp3-positive cells increased after PBS treatment (Fig. S3B), while it was limited after serum starvation. Importantly, there was no significant difference in the number of c-casp3-positive cells between control and CD90-OE MYOD1-UDCs (Fig. 3C and Table S3). Furthermore, the fusion index 14 days after *MYOD1*-induction was not improved by CD90-OE in CD90-negative MYOD1-UDCs (Fig. 3D). Matching to the immunostaining data, immunoblotting showed no difference in expression levels of MyoD1 and MyHC proteins between control and CD90-OE MYOD1-UDCs (Fig. 3E and S4A). These results indicated that CD90-OE did not affect myogenic differentiation in CD90-negative MYOD1-UDCs.

### Short hairpin CD90 RNA (shCD90) inhibited myotube formation in CD90-positive MYOD1-UDCs

**Figure 4:**
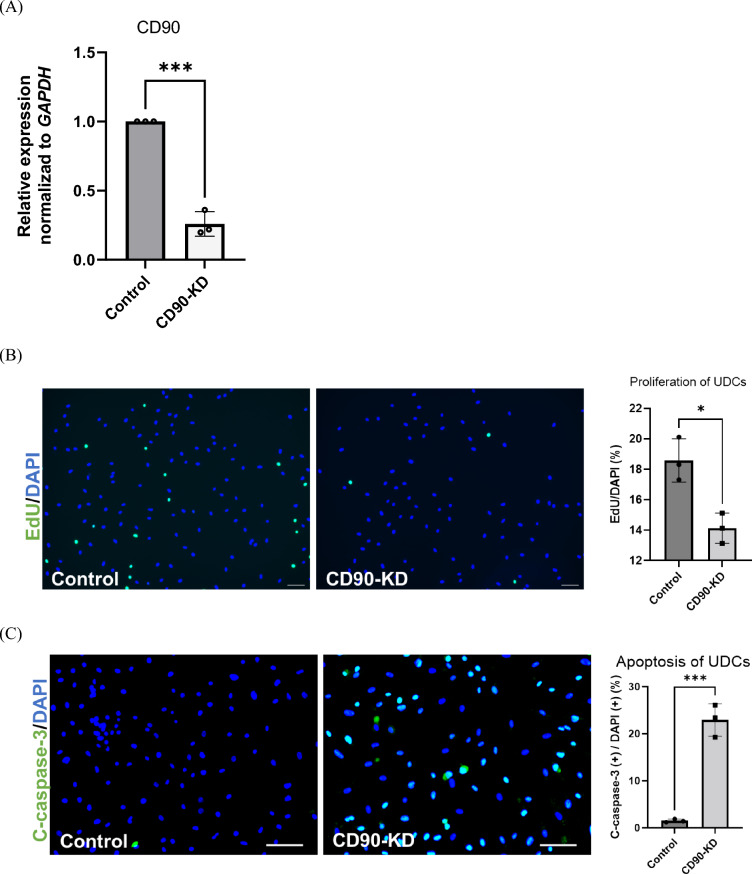

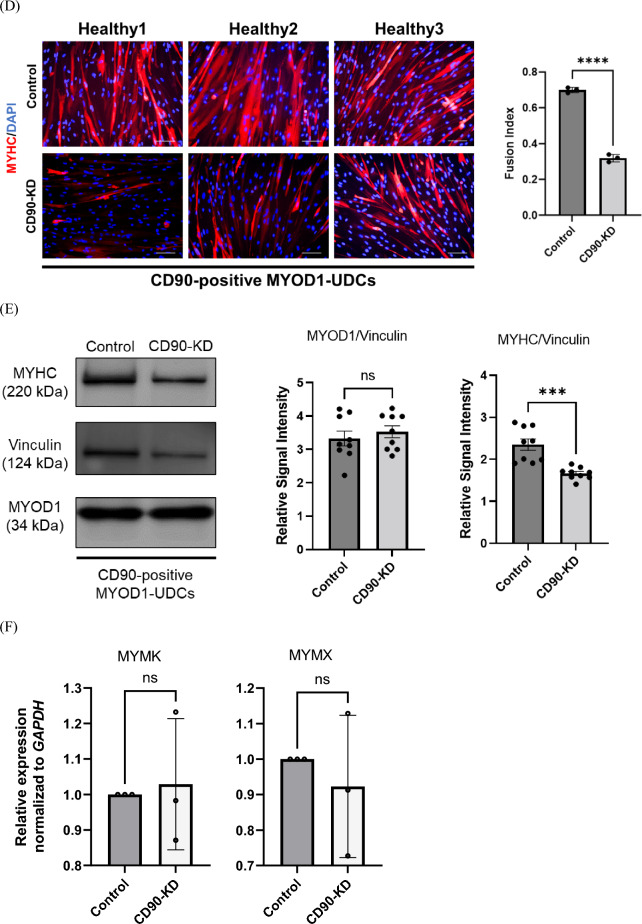
Sh*CD90* RNA inhibited CD90-positive MYOD1-UDC myotube formation. (**A**) Expression of CD90 in CD90-positive MYOD1-UDCs determined by qRT-PCR; n = 3 each. Two-tailed t-test was used for statistical analysis. Data expressed as mean ± SEM; ***P < 0.001. (**B**) EdU assay to evaluate proliferative ability of CD90-positive MYOD1-UDCs. Green, EdU; blue DAPI staining. Scale bar denotes 100 µm; n = 3 per group. Two-tailed t-test was used for statistical analysis. Data expressed as mean ± SEM; *P < 0.05. (**C**) Immunocytochemical detection of c-casp3 (green) to evaluate apoptotic ability of CD90-positive MYOD1-UDCs. Blue, DAPI staining. Scale bar denotes 100 µm; n = 3 per group. Two-tailed t-test was used for statistical analysis. Data expressed as mean ± SEM; ***P < 0.001. (**D**) Immunocytochemical detection of MyHC (red) in CD90-positive MYOD1-UDCs. Blue, DAPI staining. Scale bar denotes 100 µm. Fusion index was calculated as percentage of nuclei within MyHC-positive myotubes in nine randomly selected images from three healthy individuals. Two-tailed t-test was used for comparisons. Data expressed as mean ± SEM; ****P < 0.0001. (**E**) Immunoblotting of MyHC and MyoD1 in CD90-positive MYOD1-UDCs on 14th day after differentiation. Anti-Vinculin antibody was used as a loading control. Original blots are presented in Supplementary Figure S5A. Two-tailed t-test was used for comparisons. Data expressed as mean ± SEM; ***P < 0.001. (**F**) *MYMK* and *MYMX* expression in CD90-positive MYOD1-UDCs was detected on the 3rd day after differentiation by qRT-PCR analysis; n = 3 each. Two-tailed t-test was used for statistical analysis.

We further assessed the molecular function of CD90 during MYOD1-UDC differentiation by knockdown (KD) of *CD90* in CD90-positive MYOD1-UDCs as control using an sh*CD90* lentivirus vector and confirmed that sh*CD90* reduced approximately 70% of *CD90* expression in the cells (Fig. 4A). Cell proliferation assays showed that the number of EdU-stained proliferating cells was higher in control than in CD90-KD MYOD1-UDCs (Fig. 4B). We also evaluated the number of apoptotic cells by staining with an anti-c-casp3 antibody under serum-starved conditions, which showed that *CD90*-KD significantly increased the number of apoptotic cells in CD90-positive UDCs (Fig. 4C). Moreover, the fusion index was significantly decreased in the CD90-KD MYOD1-UDCs 14 days after the induction of myogenic differentiation (Fig. 4D). Furthermore, immunoblotting data showed that MyHC protein expression levels were reduced in CD90-KD cells following the immunostaining data (Fig. 4E and S4A). Since we could observe several changes in CD90-KD cells from the control, we performed qPCR for fusion-related genes to detect some differences. However, contrary to our expectation, CD90-KD did not affect *MYMK* and *MYMX* (Fig. 4F). CD90-KD inhibited myogenic differentiation in CD90-positive MYOD1-UDCs.

### CD90-positive MYOD1-UDC myotubes from DMD patients showed high and stable dystrophin expression during ASO treatment

**Figure 5:**
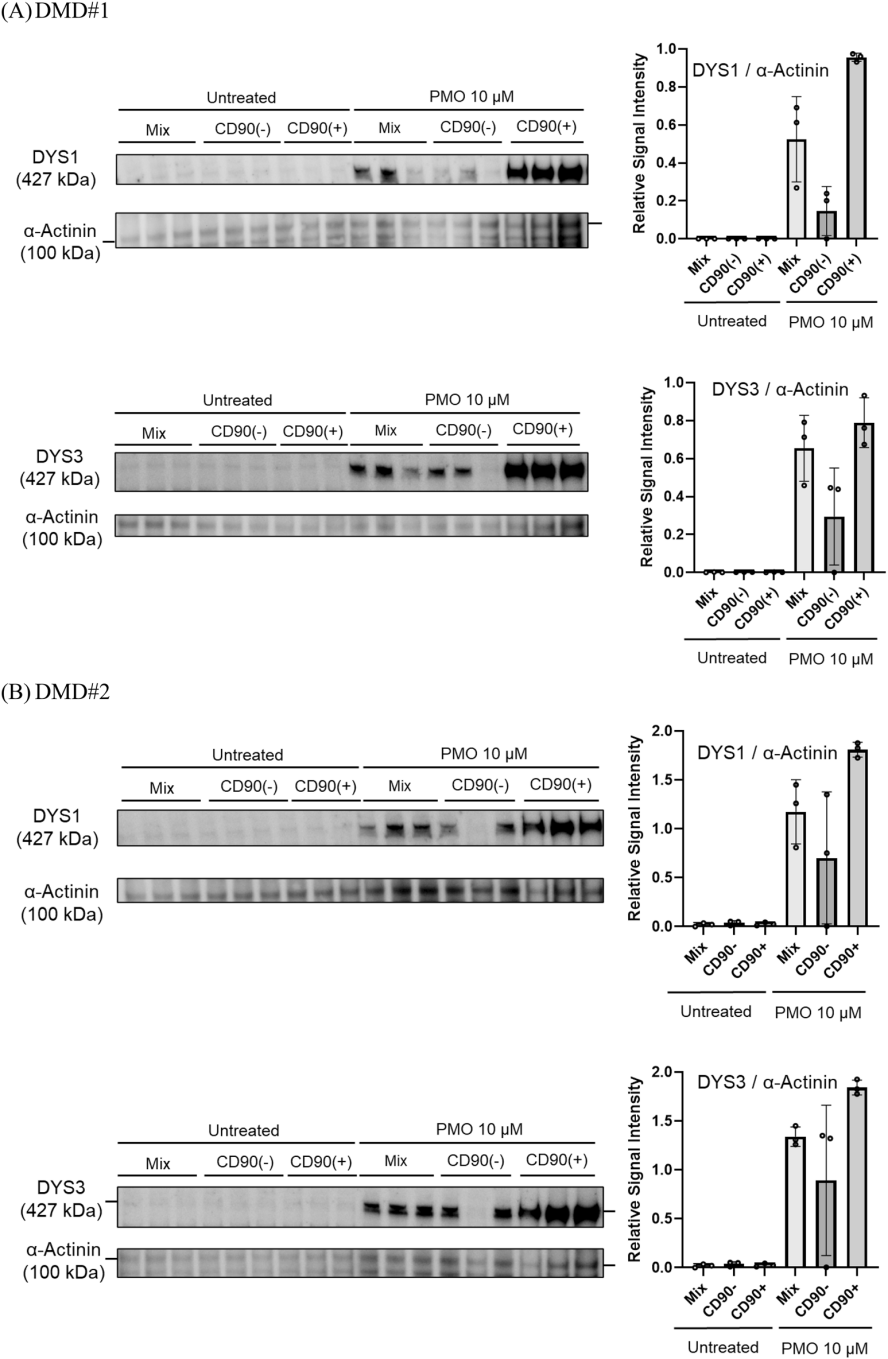

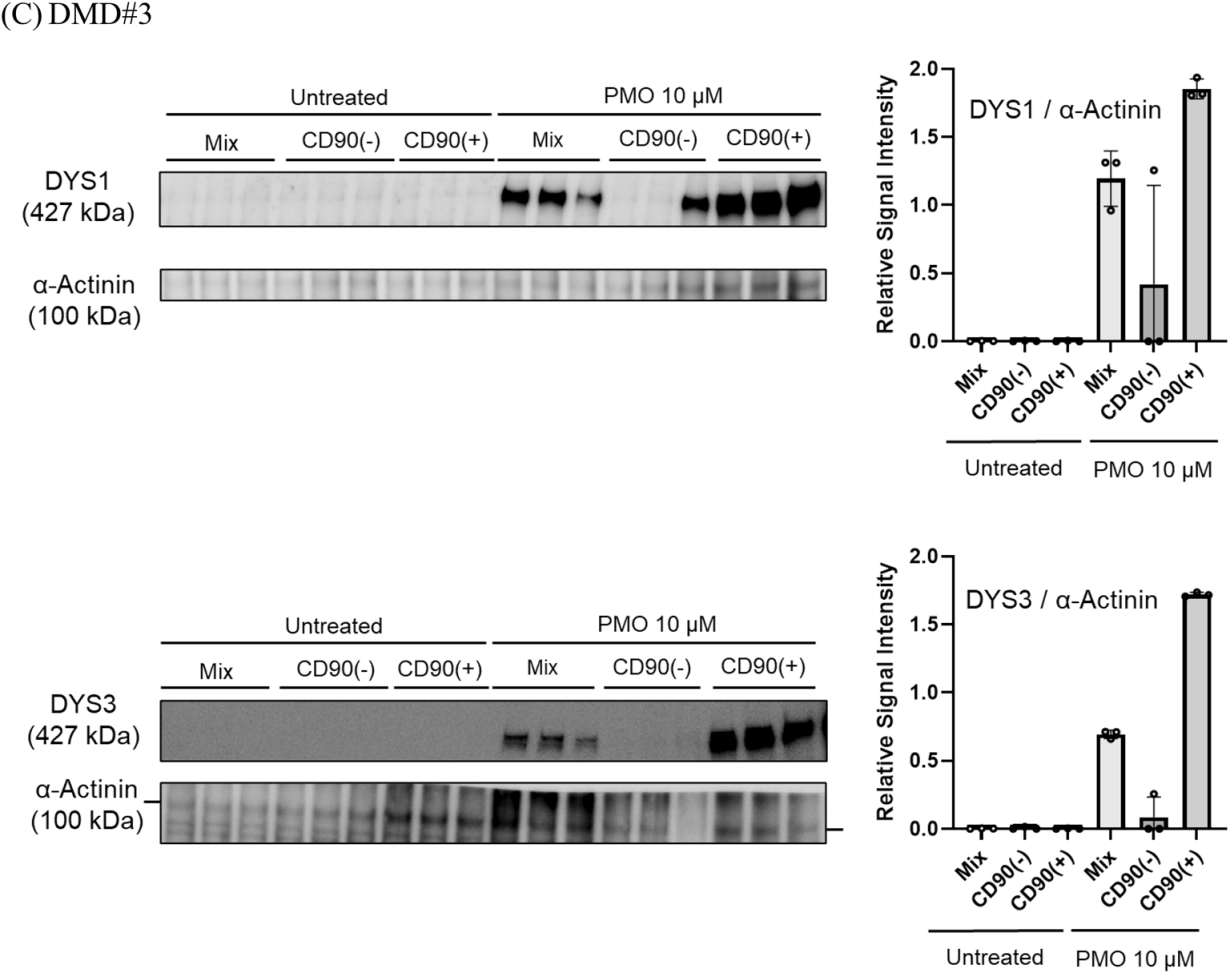
CD90-positive MYOD1-UDC myotubes from DMD patients showed high and stable dystrophin protein expression after treatment with ASOs. (**A**–**C**) Immunoblotting of dystrophin in CD90-positive, negative MYOD1-UDCs and their mix from three different DMD patients with exon 45 deletion. Graphs showed quantitative data of DYS1 and 3 expressions with and without PMO44 treatment. Data expressed as mean ± SD. Anti-α-Actinin antibody was used as a loading control. Original blots are presented in Supplementary Figure S6.

We determined the suitability of UDCs for evaluating the efficacy of ASO drugs in DMD patients by preparing UDCs from three different donors with the exon 45 deletion (DMD#1–3). The deletion in the *DMD* gene was diagnosed by multiplex ligation-dependent probe amplification, which is a reliable quantitative method for detecting deletions and duplications in all 79 exons of the *DMD* gene. Their open reading frames were restored by exon 44 skipping. We then transfected CD90-negative and CD90-positive MYOD1-UDCs and their mix with an antisense phosphorodiamidate morpholino oligomer (PMO) using Endo-Porter on seven days after differentiation and demonstrated dystrophin restoration in MYOD1-UDCs on 14 days after differentiation (Fig. 5A–C and S7A–C). The immunoblotting analysis regarding dystrophin here was confirmed by dual antibodies, including NCL-DYS1 and 3. In detail, we measured dystrophin protein levels in samples from different wells, which showed high dystrophin levels after the treatment in CD90-positive MYOD1-UDCs, with small inter-well variability (coefficient of variance after treatment detected by NCL-DYS1: 0.02 in DMD#1, 0.04 in DMD#2, and 0.04 in DMD#3; by NCL-DYS3: 0.17, 0.04 and 0.01, respectively) compared with the mix (DYS1: 0.43, 0.28, and 0.17, respectively; DYS3: 0.27, 0.07 and 0.04, respectively) and CD90-negative MYOD1-UDCs (DYS1: 0.88, 0.96, and 1.73, respectively; DYS3: 0.87, 0.86 and 1.71, respectively) (Fig. 5A–C and S7A–C).

## Discussion

Here, we report that CD90-positive UDCs comprise a limited subpopulation of UDCs with high myogenic potency. Myotubes represent relatively mature skeletal muscle models from the perspective of MRF expression and multinucleated morphology and thus have potential as muscle disease models. Indeed, CD90-positive MYOD1-UDC-derived myotubes expressed high dystrophin protein levels, which may help evaluate the efficacy of ASO treatment.

UDCs consist of heterogeneous populations of cells, and two morphologically distinct subpopulations, SS-UDCs and RS-UDCs, have been identified in UDCs from human urine samples^13^. These two subpopulations showed similar colony-forming efficiencies, but SS-UDCs demonstrated faster proliferation, higher motility, and greater potential for osteogenic and adipogenic differentiation, while RS-UDCs showed greater potential for chondrogenic differentiation. However, our data showed that SS-UDCs and RS-UDCs could change their morphologies, highlighting the need to understand the heterogeneity of UDCs from a genetic perspective. To our knowledge, this is the first study to apply scRNA-seq to UDCs. We demonstrated that UDCs comprised heterogeneous subpopulations in terms of their transcriptomic profiles. In accordance with previous reports, UDCs expressed marker genes of the upper urinary tract or MSCs. Furthermore, because the UDCs were primary cultured cells, the gene expression levels tended to differ between the two UDC samples.

CD90 is a 25 kDa cell-surface glycoprotein located in lipid rafts, which is tethered to the outer leaflet of the cell membrane by a glycosylphosphatidylinositol anchor^14^. CD90 was initially described on the plasma membrane of murine thymocytes but is also present in other cell types, including kidney glomerular mesangial cells^15^. Moreover, CD90 plays important physiological and pathological roles in development, cancer, immunity, and tissue fibrosis^16–20^. CD90 can mediate integrin-related signaling through direct interaction with integrins^14^ and has thus been considered a key adhesion molecule involved in cell–cell interaction. In our study, CD90-positive UDCs expressed several myofibroblast markers. According to previous research from other groups, injured renal epithelial cells underwent regression to the mesenchymal phenotype and then acquired a myofibroblastic nature, including expression of α-SMA^21^. They especially mentioned that the immature myofibroblast expressed CD90 highly. Furthermore, considering that CD90-positive and negative cells consistently maintained their CD90 expression characteristics during passage in the FACS analysis (Fig. 2B), both cells may represent two distinct cell types. Moreover, CD90 was shown to be a versatile modulator of signaling affecting cellular proliferation and survival^22,23^. CD90 promotes the proliferation of mesangial cells via the activation of Smad1 and STAT3 and the proliferation of T cells through a pathway involving calcineurin, protein tyrosine kinases, phosphoinositide-3 kinase, protein kinase C, and mitogen-activated protein kinase^22^. Additionally, the apoptosis-related genes *NGFI-B* and *GADD45G* were upregulated in an anti-CD90-induced model of glomerulonephritis^23^. In the present study, CD90-knockdown inhibited proliferation and promoted apoptosis of UDCs, but there was no difference in either property between CD90-positive and CD90-negative UDCs, suggesting that a threshold level of CD90 expression might be required to affect proliferation and apoptosis.

CD90 expression has been reported during muscle development in rats, mice, and human myoblasts in vivo and in vitro^24–26^. These studies reported that CD90 was present on myoblasts and, in some cases, on early myotubes but was absent from mature myotubes. This evidence for the developmental regulation of CD90 and the finding of a non-fusing variant of rat myoblasts^24^ that did not express CD90 suggest that CD90 might be involved in the fusion process. The current data accordingly demonstrated that CD90 expression decreased during MYOD1-UDC differentiation to myotubes. Based on the observation that sh*CD90* decreases the fusion index of MYOD1-UDCs, it is suggested that CD90 could be involved in cell fusion. Separately, MYMK and MYMX are known to regulate specific processes in membrane remodeling during myoblast fusion: MYMK is critical for integrating the outer lipid monolayer of plasma membranes, while the ectodomains of MYMX are responsible for fusion pore development through the induction of membrane stresses^27^. Nevertheless, in this context, sh*CD90* did not influence the expression levels of MYMK and MYMX.

MyoD1 is regarded as a master MRF that regulates myogenic differentiation^28^. Following the discovery of MYOD1-mediated direct reprogramming in fibroblasts, Weintraub et al. investigated its ability to convert various initial cell types, including adipocytes, neuroblastomas, and liver cells, into myogenic cells^29^. *MYMK, MYMX, MYOG*, and *MYHC* are regulated under the control of MyoD1^28,30^. Regarding UDCs, MyoD1 needs other epigenetic factors to convert them to the skeletal muscle lineage. We previously suggested that reprogramming terminally differentiated UDCs by epigenetic treatment could be sufficient to achieve lineage conversion^12^. Our method using the histone methyltransferase inhibitor DZNep significantly enhanced myogenin expression in MYOD1-UDCs, leading to multinucleated myotubes that expressed adequate levels of muscle-related proteins, including MyHC and dystrophin. However, we noted that other factors, besides regulation of histone methylation by DZNep, were still needed to promote myogenesis of MYOD1-UDCs and considered that unknown factors in CD90-positive UDCs might explain this.

CD90-positive MYOD1-UDC myotubes derived from DMD patients provided highly reliable and reproducible data regarding the efficacy of ASO drugs based on their high and stable dystrophin expression levels. CD90-positive MYOD1-UDCs may thus improve on current in vitro human muscle models, enabling a more predictive screening strategy for the preclinical selection of effective new ASOs earlier in the drug discovery process.

We recognize several remaining issues that require further investigation to evidence our hypothesis that CD90-positive UDCs have the potential to differentiate into myogenic cells. First, we also need to perform additional scRNA-req with MYOD1-UDCs from the same healthy donor. We consider it valuable to confirm CD90-positive UDCs differentiate into myogenic cells by tracing cell fate using trajectory analyzing method^31^. Second, we need to clarify the precise mechanism underlying the regulation of *MYMK, MYMX, MYOG*, and *MYHC* expression with *MYOD1* in CD90-positive UDCs. Since we speculate that other unknown factors function parallelly in CD90-positive MYOD1-UDCs, we are currently searching for genes as candidates which express highly on CD90-positive population in our scRNA-seq.

In conclusion, UDCs comprise heterogeneous subpopulations, and specific subpopulations expressing CD90 have the high potential to differentiate into myotubes with *MYOD1* induction. Since CD90 itself does not promote differentiation, other factors assisting MYOD1 function may be crucial for differentiating CD90-positive MYOD1-UDCs. By solving the mysteries in UDCs, we can likely elucidate unknown mechanisms of general myogenic differentiation in humans. Moreover, immunoblotting using CD90-positive MYOD1-UDCs from DMD patients with exon 45 deletion should support detecting stably high intense bands of dystrophin under treatment of the exon 44 skipping ASO. Given this fact, we expect our system to be applied to various DMD patients with other mutations in future ASO drug development.

## Methods

### Ethics statement

This study was approved by the Ethics Committee of the National Center of Neurology and Psychiatry (Approval ID: A2018-029), and we obtained informed consent from all donors before collecting urine. During all experiments, we followed the relevant guidelines and regulations. Moreover, the characteristics of all donors are shown in Supplementary Table 1.

### Isolation and culture of UDCs

We collected urine samples with sterilized plastic bottles and isolated UDCs according to a previously published protocol^32^. Briefly, we centrifuged urine samples at 400 × *g* for 10 min at 15–25 °C. Acquired cell pellet was resuspended in growth medium consisting of a 1:1 mix of high-glucose Dulbecco's Modified Eagle Medium (DMEM) and REGM Bullet Kit (Lonza, Switzerland) supplemented with 15% tetracycline-free fetal bovine serum (FBS), 0.5% GlutaMAX-I (Gibco), 0.5% nonessential amino acids (Thermo Fisher Scientific), basic fibroblast growth factor (Sigma, St Louis, MO, USA), platelet-derived growth factor-AB (Peprotech, Rocky Hill, NJ, USA), epidermal growth factor (Peprotech), amphotericin B and penicillin/streptomycin (P/S). Cells were seeded in gelatin-coated plates and cultured at 37 °C and 5% CO_2_ for 3 days. The medium was replaced with fresh one on day 4 and changed every other day.

### Flow cytometry

Flow cytometric evaluation of UDCs was performed using fluorescein isothiocyanate-conjugated anti-human CD90 antibody (BioLegend, #328107) and 7-AAD (BD Biosciences, 51-68981E). UDCs were trypsinized and washed with growth medium, centrifuged at 200 × *g* for 5 min, collected, and suspended in phosphate-buffered saline (PBS) with 2% FBS. The antibody was added to UDC suspension at an optimal concentration (1:200) and incubated for 30 min on ice in the dark. Moreover, the 7-AAD was added to UDC-suspension at an optimal concentration (1:100) and incubated for 10 min before analysis. The cells were centrifuged, resuspended in PBS with 2% FBS, passed through a 70 μm filter, and analyzed using a SONY FACS SH800S (Sony Biotechnology, San Jose, CA, USA) and Cell Sorter Software (Sony).

### Myogenic differentiation of UDCs

We performed direct reprogramming of UDCs into myotubes, as described previously^12^. For context, we transduced *MYOD1* into UDCs using a retroviral vector containing a doxycycline-inducible *MYOD1* expression system at MOI of 200 in 60 mm gelatin-coated plates. The construct of this vector can be found in our previous article^12^. Additionally, we differentiated MYOD1-UDCs into myotubes by switching the growth medium to the differentiation medium with doxycycline and 5 μM DZNep. The differentiation medium was replaced with fresh one without DZNep after 3 days, and the medium was changed every 3 days after that.

### ScRNA-seq

To obtain scRNA-seq data from Myo- and nonMyo-UDCs, we used Chromium Next GEM Single Cell Multiome Reagent Kits (10 × Genomics) according to the manufacturer's instructions. Briefly, single nuclei were isolated from cultured cells and resuspended in diluted buffer. Using a microfluidic chip, the nuclei are partitioned into Gel Beads-in-emulsion (GEM). In each GEM, primers containing an Illumina TruSeq Read 1, 16 nt 10 × Barcode, 12 nt unique molecular identifier (UMI), and a 30 nt poly(dT) sequence were released and mixed with mRNA and reverse transcription reagents. Incubation of the GEMs produced 10 × Barcoded, full-length cDNA from poly-adenylated mRNA. This cDNA was amplified via PCR to construct library with sufficient mass of gene expression. Afterwards, the cDNA library was run on an Illumina Novaseq 6000 (Illumina). The sequenced reads were converted into FASTQ files using the Cell Ranger pipelines provided by 10 × Genomics with the hg38 human reference genome. Using the Seurat package (v.4.0.1)^33^, we removed low-quality reads and PCR duplicates from further analyses. We filtered out low-quality cell data by the threshold of 1000–100,000 UMI per cell, > 3000 genes per cell, and < 10% mitochondrial gene expression. Consequently, 6,518 and 10,974 cells were used for Myo- and nonMyo-UDC experiments, respectively. Using the Monocle3^34^, we performed uniform manifold approximation and projection (UMAP) analysis to cluster each cell based on gene expression and annotated each cluster by characteristic genes.

### RNA overexpression and knockdown

CD90-overexpression lentiviral plasmids (sequence: NM_001311162.2) and shRNA lentiviral plasmids targeting CD90 (target sequence: CACCAGCAAATACAACATGAA) were purchased from VectorBuilder (Tokyo, Japan). We transfected lentiviral vectors and packaging plasmids (MDL/RRE, Rev, and VSV-G) into HEK 293T cells with Lipofectamine 3000 (Invitrogen). Additionally, we collected the viral supernatants 2 days after transfection and concentrated them with Lenti-X lentivirus concentrator (Clontech). To titrate those lentiviral supernatants, we used a Lenti-X p24 titer kit (Takara). We transfected them to MYOD1-UDCs following multiplicity of infection of 100. MYOD1-UDCs were exposed to the growth medium with lentivirus for 48 h and selected by culturing in the growth medium with 10 µg/mL blasticidin (Wako).

### Antisense PMO transfection

PMO targeting exon 45 was synthesized by Gene Tools (Philomath, OR, USA). We transfected this PMO (10 μM final concentration) into MYOD1-UDCs from DMD patients on the 7th day after differentiation using the Endo-Porter transfection reagent (Gene Tools, Philomath, OR, USA). After 72 h incubation with the PMO, the medium was changed to fresh PMO-free differentiation medium.

### RNA analysis

Total RNA was extracted from UDCs and MYOD1-UDCs using an RNeasy RNA isolation kit (Qiagen). From the RNA, cDNA was produced using a Go-script Reverse Transcription Kit (Promega). Afterwards, we mixed the cDNA with the GoTaq qPCR Master Mix (Promega). The specific primers used for qPCR are listed in Supplementary Table 2. The mRNA expression levels were quantified using an ABI StepOne real-time PCR machine (Thermo Fisher Scientific) using the comparative Ct (ΔΔCt) method^35^.

### Protein extraction and immunoblotting analysis

Total protein extraction was prepared from cultured cells using RIPA buffer supplemented with cOmplete Mini Protease Inhibitor Cocktail (Roche, Meylan, France). We sonicated the lysates with 10 pulses for 10 s in cold water using SONIFIER (BRANSON 250D; Central Science Trade Co. Ltd., Tokyo). The sonicated lysates were centrifuged at 15,000 × *g* for 15 min at 4 °C. We collected the supernatant and measured protein concentrations by using a BCA protein assay kit (Thermo Fisher Scientific). Protein samples mixed with NuPAGE LDS Sample Buffer (Thermo Fisher Scientific) were denatured at 70 °C for 10 min. With those samples, we performed electrophoresis using NuPAGE Novex Tris–Acetate Gel 3–8% (Invitrogen) at 150 V for 60 min. Afterwards, proteins on the gel were transferred to polyvinylidene difluoride (PVDF) membranes. The membranes were then blocked with 5% ECL Prime blocking agent (Cytiva) in PBS containing 0.1% Tween 20 for 1 h at room temperature, subsequently incubated with primary antibodies diluted in PBS-T overnight at 4 °C. The following primary antibodies were used for immunoblotting: anti-MYOD1 (1:200; Santa Cruz, sc-32758), anti-MyHC (1:200; eBioscience, #14-6503-82), anti-dystrophin (1:20; Leica, NCL-DYS1, DYS2 and DYS3), anti-α-Actinin/ACTN1 (1:250; Abcam, ab68194), anti-α-tubulin (1:1200; Sigma, T5168), anti-GAPDH (1:1000; Sigma, MAB374) and anti-Vinculin (1:1000; Abcam, ab91459). The PVDF membranes were then washed with PBS-T and incubated with anti-mouse and rabbit horseradish peroxidase-conjugated secondary antibodies (1:10,000; Cytiva, NA9310 and 9340) in PBS-T for 1 h at room temperature. The signals were detected by ECL Prime Western Blotting Detection Reagent (GE Healthcare, Buckinghamshire, UK) using the ChemiDoc MP imaging system (Bio-Rad, Hercules, CA, USA). We quantified the acquired signals using Image Lab software (Bio-Rad). We used Restore™ Plus Stripping Buffer (Thermo Fisher Scientific) for stripping antibodies with 5 min of incubation.

### Time-lapse imaging

UDCs from a healthy 30-year-old male were cultured and observed continuously by microscopy. Time-lapse images were analyzed using the integrated software package TI Workbench^36^.

### Immunofluorescence microscopy

Cultured cells were fixed with 4% formaldehyde for 10 min at 4 °C, subsequently permeabilized in 0.1% triton-X (MP Biomedicals, USA) for 5 min at room temperature and blocked with PBS containing 10% goat serum for 1 h at 37 °C. Incubations with primary antibodies to MyHC (1:200; eBioscience, #14-6503-82), c-casp3 (1:200; Cell Signaling Technologies, Danvers, MA, USA #9664), and MYOD1 (1:200; Santa Cruz, sc-32758) were performed overnight at 4 °C. After washing with PBS, they were incubated with a secondary antibody conjugated with Alexa-488 or -546 (1:300; Molecular Probes) for 30 min at room temperature. Following the manufacturer's instructions, we performed EdU staining with a Click-iT EdU imaging kit (Invitrogen) and stained nuclei with 4,6-diamidino-2-phenylindole (DAPI). We analyzed the stained cells using a BZ-X810 fluorescence microscope (Keyence, Osaka, Japan). We calculated the fusion index as a percentage of nuclei within MyHC-positive myotubes in nine randomly selected images from three healthy individuals.

### Statistical analysis

Statistical analyses were done by GraphPad Prism Version 9.2.0 (GraphPad Software, San Diego, CA, USA). All quantitative data (except Fig. 5 and S6) were presented as mean ± standard error. To calculate the coefficient of variance (= standard deviation/ mean), data in Fig. 5 and S6 were presented as mean ± standard deviation. We applied two-tailed t-tests for comparisons between the two groups. Throughout this article, we considered *P* value < 0.05 as statistically significant.

### Supplementary Information

Supplementary Information 1.

Supplementary Movie 1.

## Data Availability

All data from the present study are included in this article and the supplementary information file.
