# Supplementary material for: Characterization of CD90/Thy-1 as a crucial molecular signature for myogenic differentiation in human urine-derived cells through single-cell RNA sequencing
Source: Sci Rep. 2024 Jan 28;14:2329. doi: 10.1038/s41598-024-52530-5 (PMC10822841; doi:10.1038/s41598-024-52530-5)
Supplement: Supplementary file 1 — Supplementary Information 1. [file 41598_2024_52530_MOESM1_ESM.pdf]

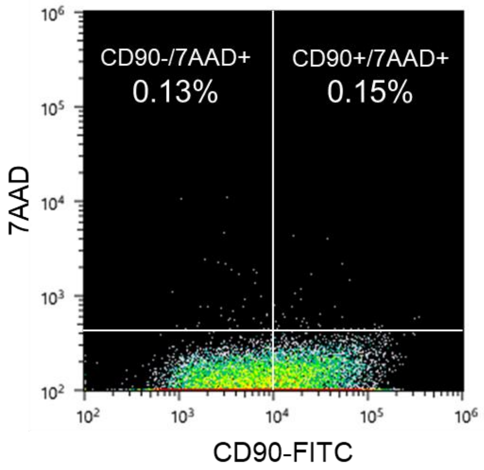

Supplementary Figure S1. Vitality of CD90-positive and negative UDCs. Representative flow cytometric analysis of CD90 and 7AAD in UDCs at 3rd passage.

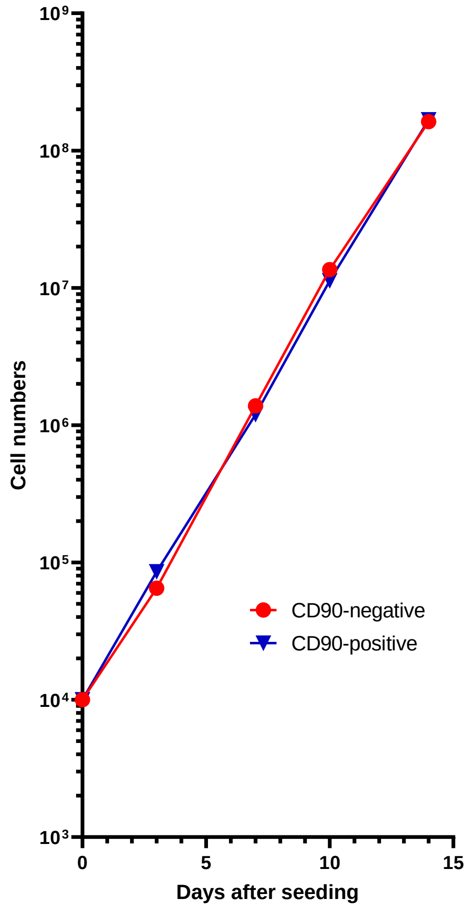

Supplementary Figure S2. Cell proliferation curves of CD90-positive and negative UDCs. 10^4 cells of CD90-positive and negative UDCs were seeded on gelatin-coated plates. On days 3, 7, 10 and 14, the cultured cells were trypsinized and calculated manually with microscopy.

(A)

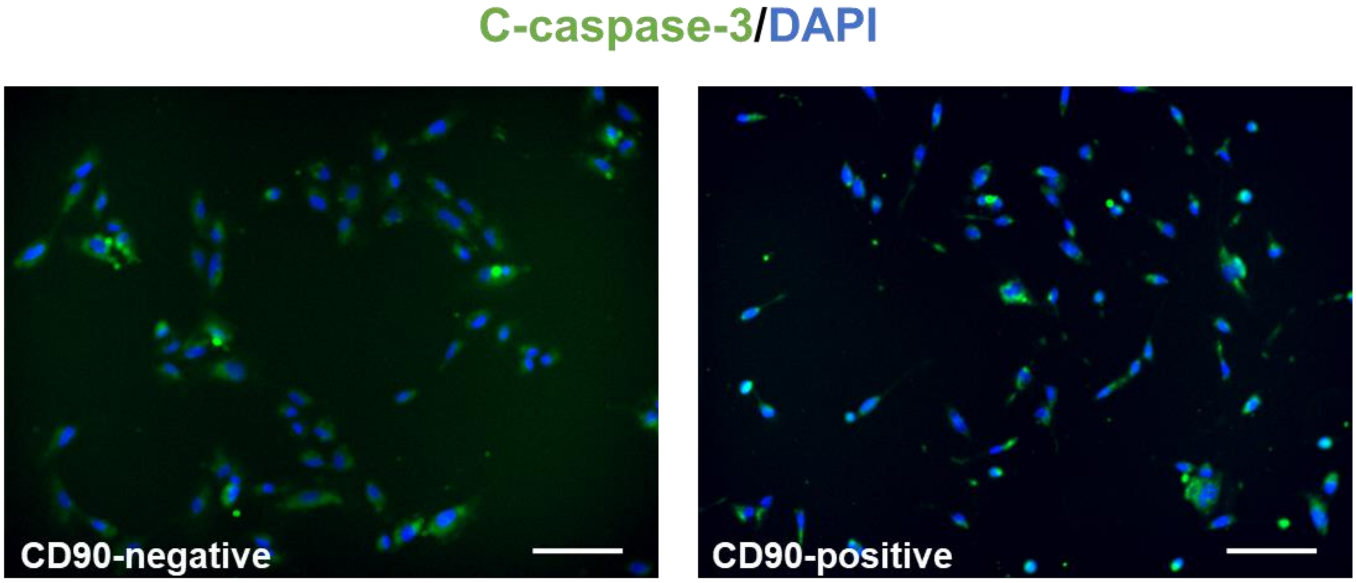

(B)

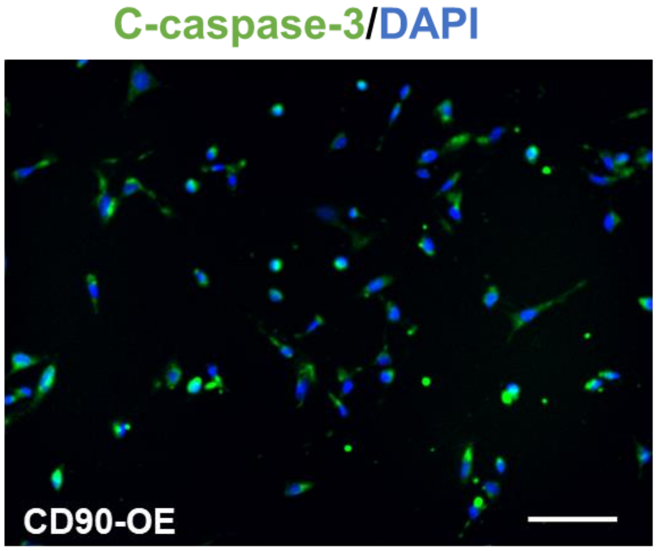

Supplementary Figure S3. Immunocytochemical detection of c-casp3 in UDCs under strict starved conditions. (A)Representative images of c-casp3-positive cells in CD90-negative and -positive MYOD1-UDCs incubated with PBS for 16h. (B)Representative images of c-casp3-positive cells in CD90-overexpressed CD90-negative (described as “CD90-OE”) MYOD1-UDCs incubated with PBS for 16h.

(A)

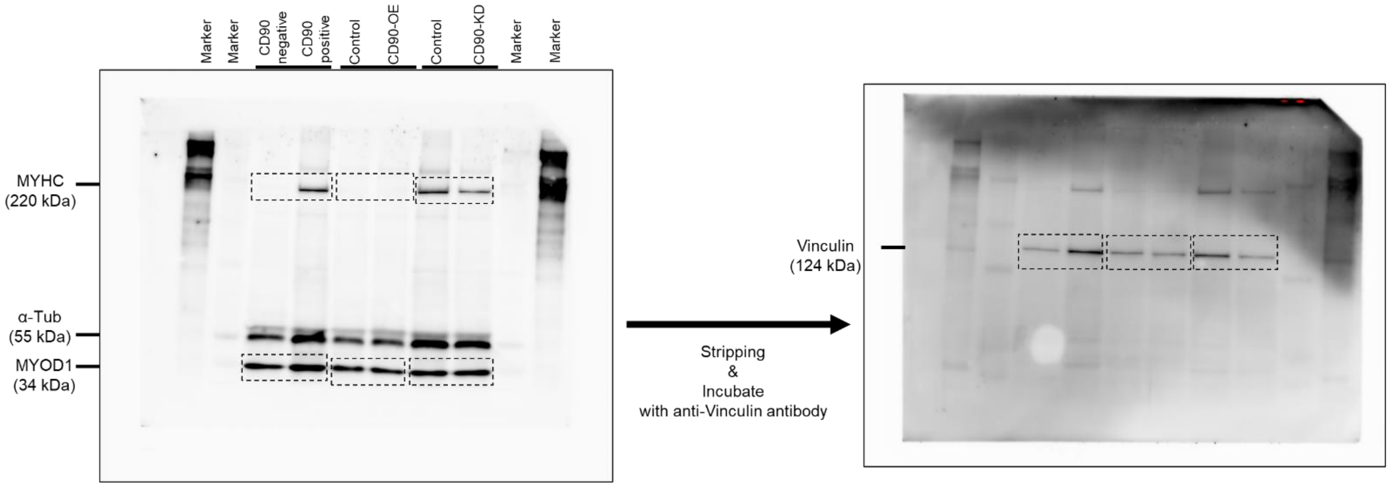

(B)

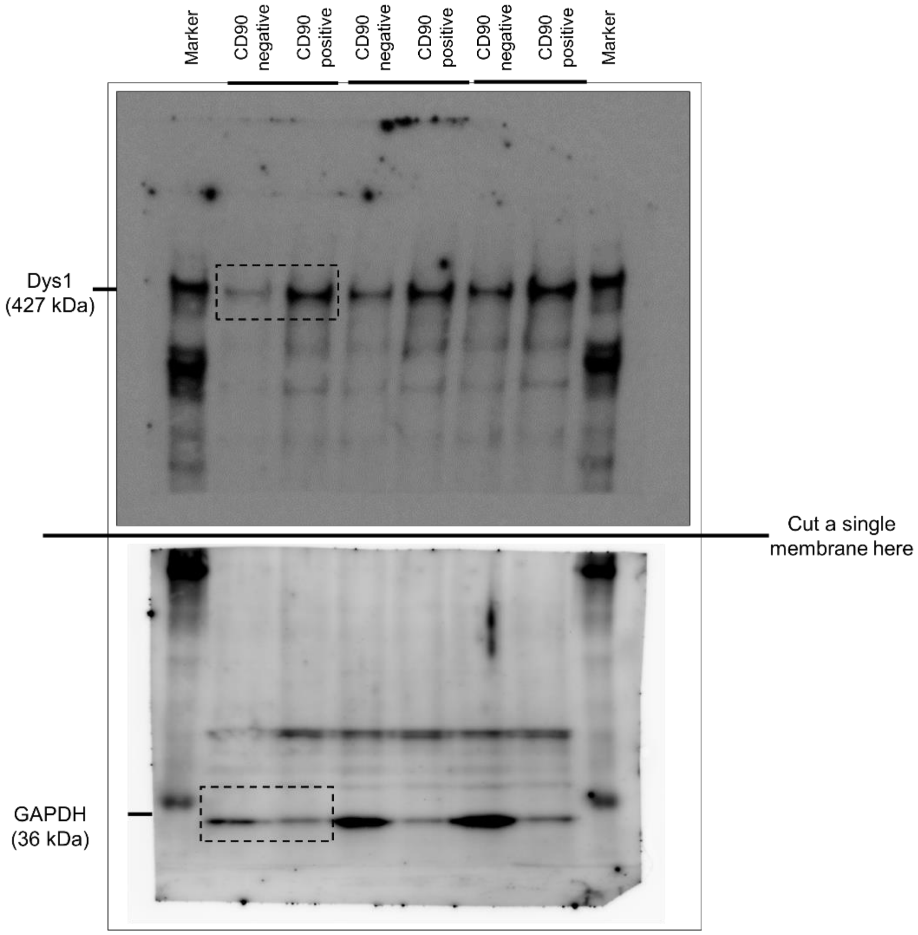

Supplementary Figure S4. Immunoblotting analysis for MYOD1, MyHC and dystrophin using MYOD1-UDCs from healthy individuals. (A) Image of the whole membrane of MYOD1 and MyHC staining in MYOD1-UDCs. Left 2 wells presented CD90-negative and -positive MYOD1-UDCs; Cropped images marked with dot lines were shown in Fig. 2F. Middle 2 wells presented CD90-negative (described as “Control”) and CD90-overexpressed CD90-negative (described as “CD90-OE”) MYOD1-UDCs; Cropped images marked with dot line were shown in Fig. 3E. Right 2 wells presented CD90-positive (described as “Control”) and CD90-knocked down CD90-positive (described as “CD90-KD”) MYOD1-UDCs; Cropped images marked with dot lines were shown in Fig. 4E. Anti-α-Tubulin and Vinculin antibodies were used as a loading control. (B) Images of whole membranes of dystrophin (DYS1) in CD90-negative and -positive MYOD1-UDCs; Cropped images marked with dot lines were shown in Fig. 2G. An anti-Vinculin antibody was used as a loading control.

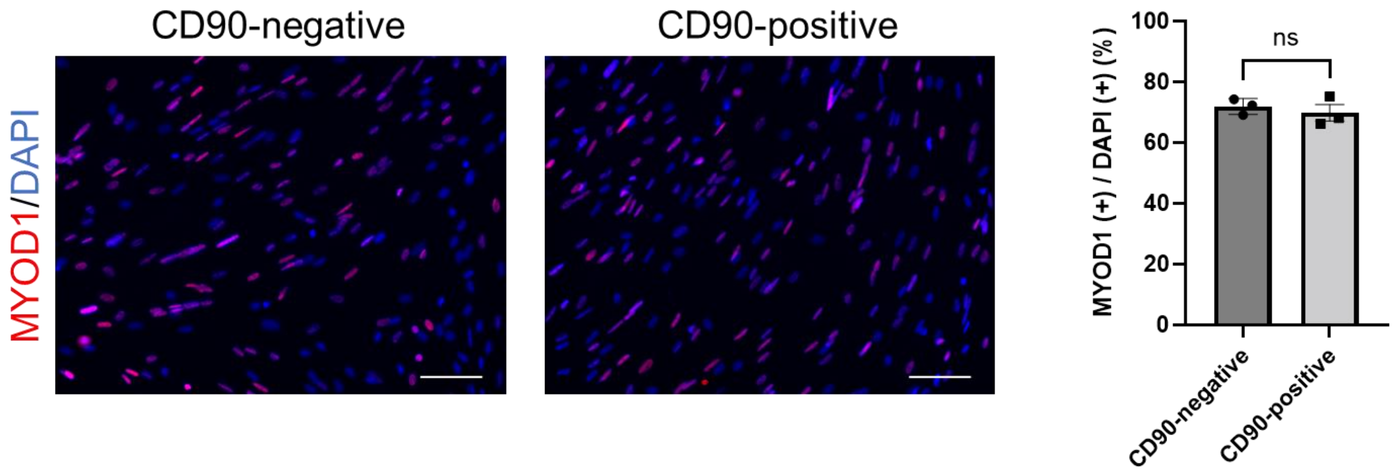

Supplementary Figure S5. Transduction of *MYOD1* retroviral vector into CD90-positive and negative UDCs at MOI of 200. The percentage of MYOD1-positive cells in CD90-positive and negative UDCs on Day 2 was 71.9 and 69.83%, respectively. Two-tailed t-test was used for statistical analysis. Error bar is SEM, n = 3. Scale bar: 100 μm.

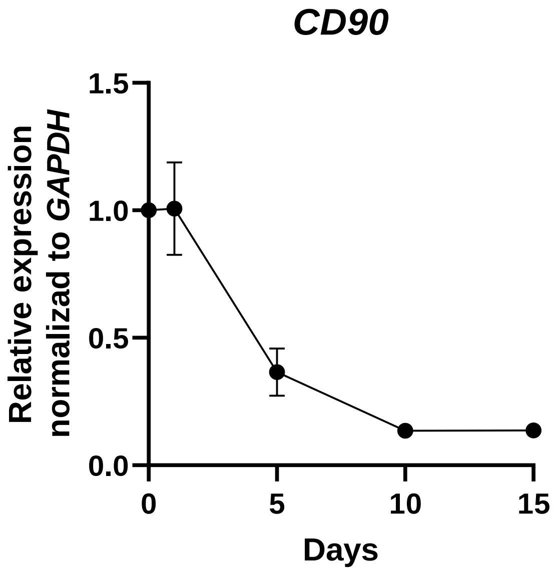

Supplementary Figure S6. Temporal profile of CD90 expression in differentiated CD90-positive MYOD1-UDCs. CD90 gene expression levels after differentiation were detected by qRT-PCR, with glyceraldehyde 3-phosphate dehydrogenase (*GAPDH*) as an internal control; n = 3 each. Data expressed as mean ± SEM.

(A) DMD#1 with DYS1 and DYS3

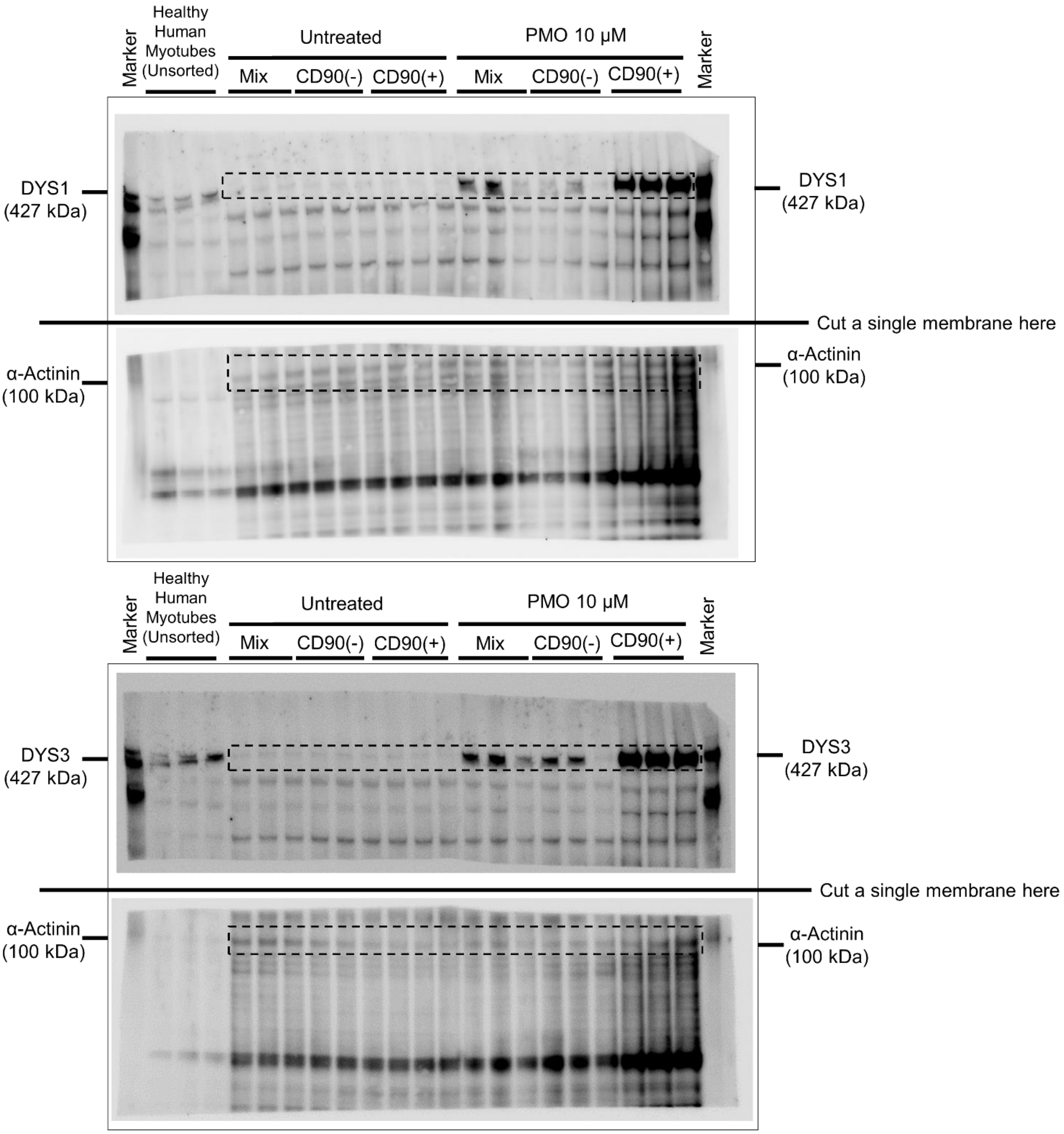

(B) DMD#2 with DYS1 and DYS3

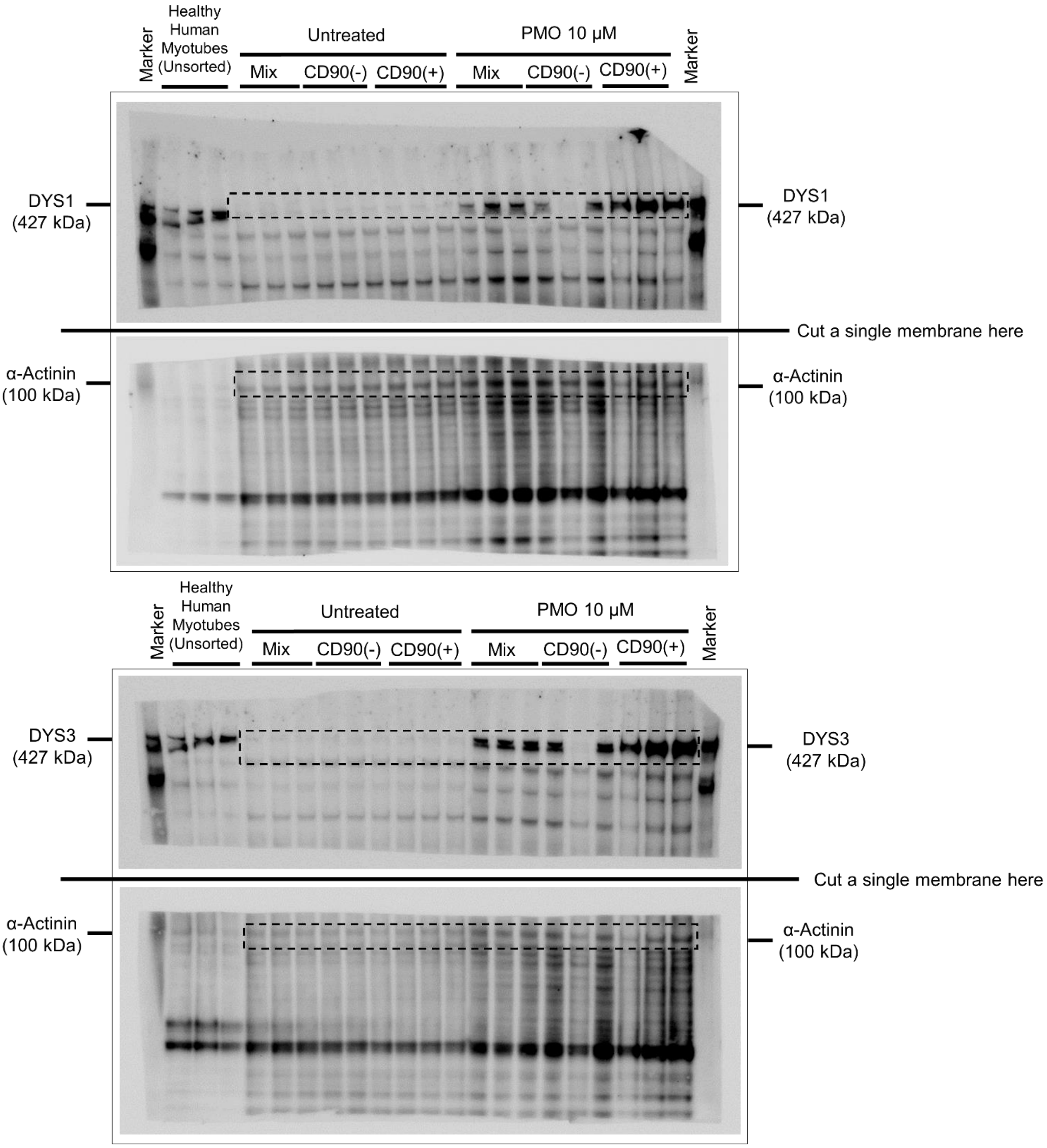

(C) DMD#3 with DYS1 and DYS3

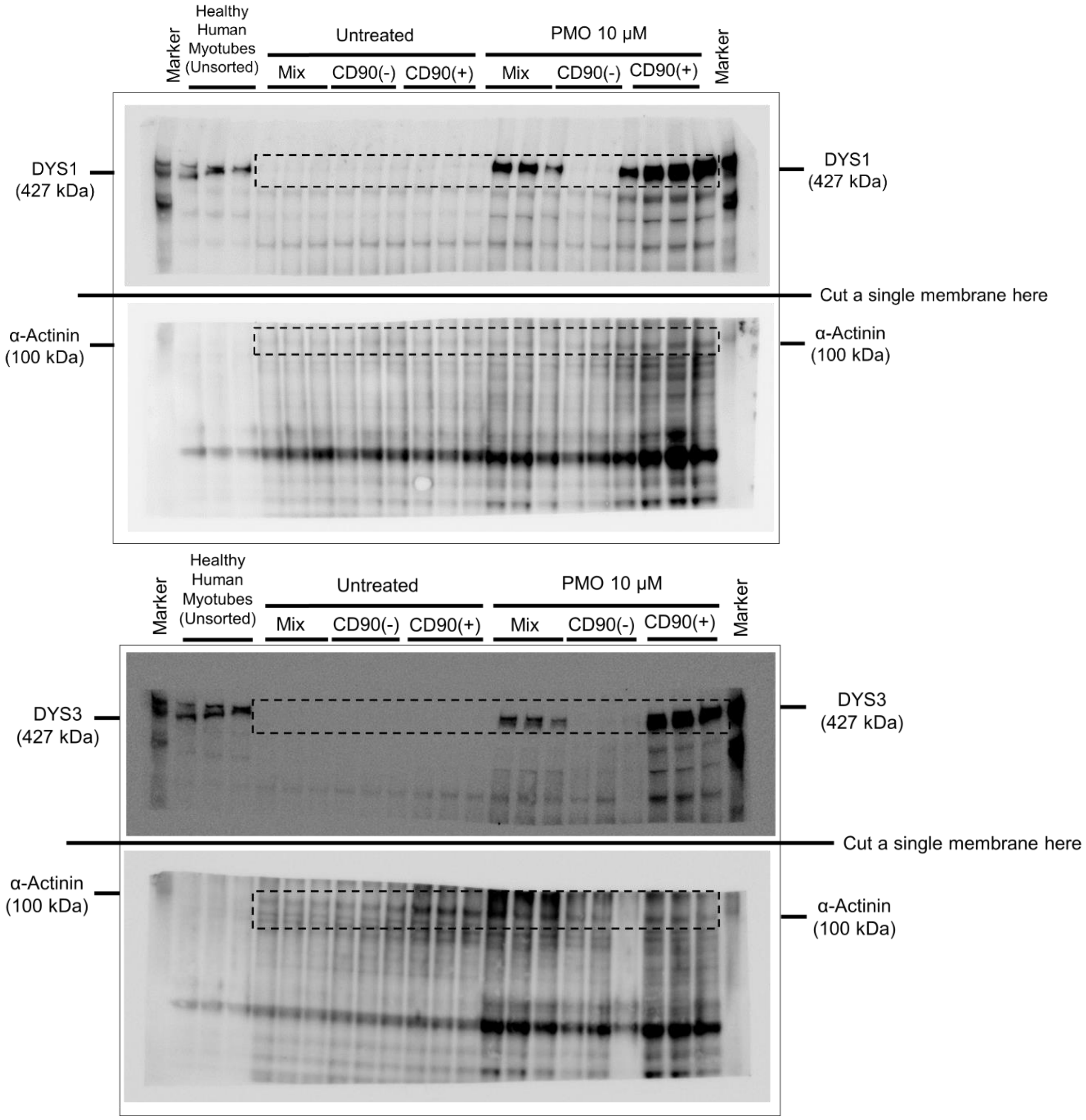

Supplementary Figure S7. Immunoblotting analysis for dystrophin using MYOD1-UDCs from DMD patients with exon 45 deletion. (A-C) Images of whole membranes of dystrophin (DYS1 and 3) in CD90-negative, CD90-positive MYOD1-UDCs and their mix from three different DMD patients (DMD#1-3) with and without PMO 44 treatment. An anti-α-Actinin antibody was used as a loading control. Healthy Human Myotubes (Lonza; CC-2580), not sorted by anti-CD90 antibody, were loaded one-tenth of MYOD1-UDCs to 3 left wells as reference. Cropped images marked with dot lines are shown in Fig. 5.

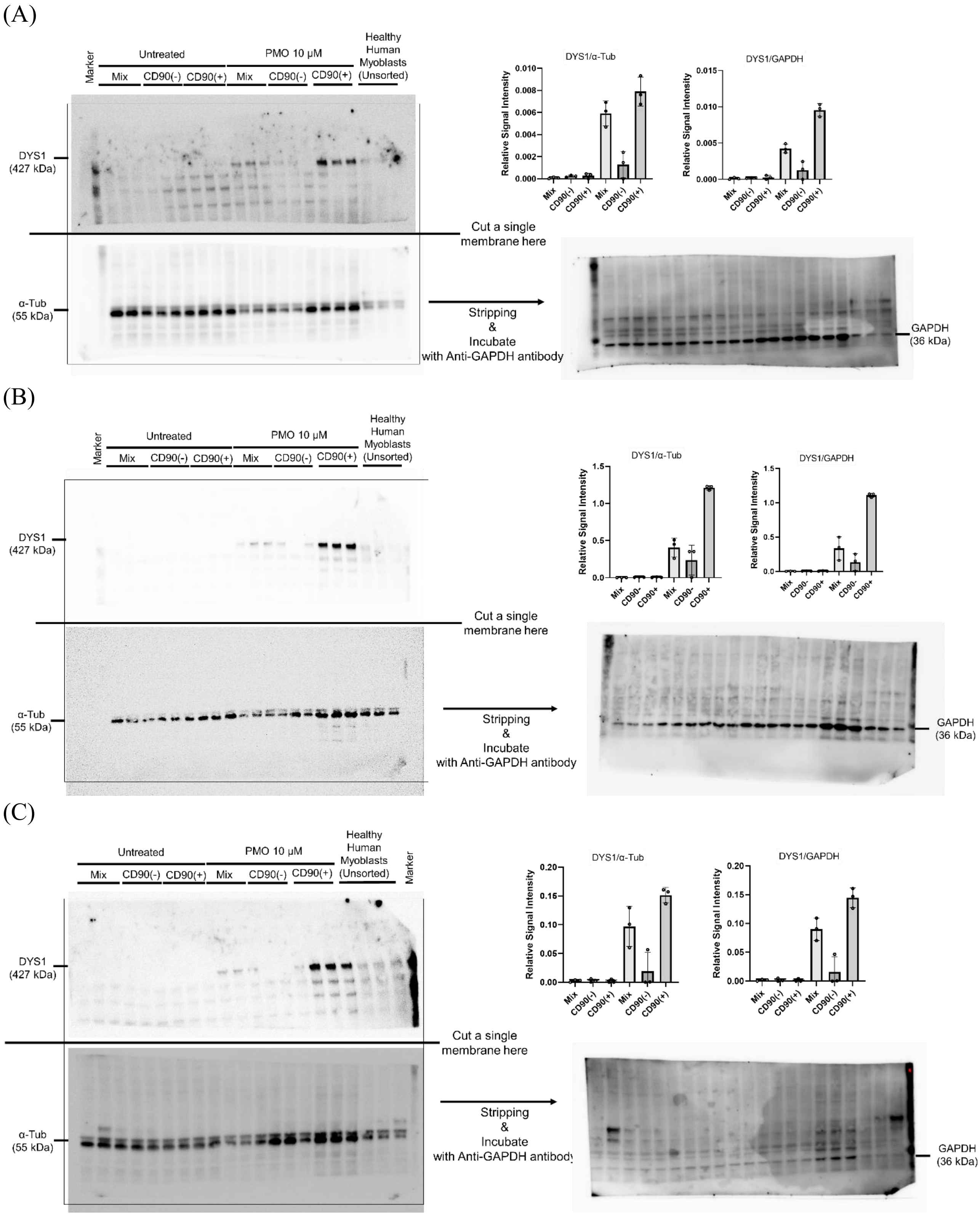

Supplementary Figure S8. Immunoblotting analysis for dystrophin using MYOD1-UDCs from DMD patients with exon 45 deletion. (A-C) Images of whole membranes of dystrophin (DYS1) in CD90-negative, CD90-positive MYOD1-UDCs and their mix from three different DMD patients (DMD#1-3) with and without PMO 44 treatment. Anti-α-Tubulin and GAPDH antibodies were used as a loading control. Data expressed as mean ± SD. Healthy Human Myotubes (Lonza; CC-2580), not sorted by anti-CD90 antibody, were loaded to the three rightmost wells adjacent to the marker for reference.

Healthy Subjects		
**ID**	**Sex**	**Age**
1	Male	30
2	Male	32
3	Female	28

DMD Patinets (exon 45 deletion)		
**ID**	**Sex**	**Age**
DMD#1	Male	4
DMD#2	Male	10
DMD#3	Male	13

Supplementary Table 1. Characteristics of UDC donors.

Quantitative PCR (qPCR) Primers		
**Genbank Number**	**Primer Name (Human)**	**Sequence (5'-3')**
NM_002046	GAPDH_Forward	ACCACAGTCCATGCCATCAC
	GAPDH_Reverse	CCACCACCCTGTTGCTGTAG
NM_001080483	MYMK_Forward	ATGCGTCACGACATCCTGGAGT
	MYMK_Reverse	CAATGGTCAGGACGCCGAACAT
NM_001315494	MYMX_Forward	CTCCCAAAGACCACTCCTAATC
	MYMX_Reverse	TGTCACTTGTCCCTCAGTTAAG
NM_006288	CD90_Forward	GGACATTGGGAAGCATCCTTGG
	CD90_Reverse	TGGCTTCCCTCTTCACGAACTC

Supplementary Table 2. List of primers used for qRT-PCR.

Movie 1. Time-lapse imaging of cultured UDCs. Differently shaped UDCs move around the plates and change their morphology from rice to spindle or spindle to rice.
